# Enhancing Recombinant Protein Yields in the *E. coli* Periplasm by Combining Signal Peptide and Production Rate Screening

**DOI:** 10.3389/fmicb.2019.01511

**Published:** 2019-07-23

**Authors:** Alexandros Karyolaimos, Henry Ampah-Korsah, Tamara Hillenaar, Anna Mestre Borras, Katarzyna Magdalena Dolata, Susanne Sievers, Katharina Riedel, Robert Daniels, Jan-Willem de Gier

**Affiliations:** ^1^Department of Biochemistry and Biophysics, Center for Biomembrane Research, Stockholm University, Stockholm, Sweden; ^2^Institute of Microbiology, University of Greifswald, Greifswald, Germany

**Keywords:** *Escherichia coli*, recombinant protein, periplasm, signal peptide, protein production rate, protein production screen

## Abstract

Proteins that contain disulfide bonds mainly mature in the oxidative environment of the eukaryotic endoplasmic reticulum or the periplasm of Gram-negative bacteria. In *E. coli*, disulfide bond containing recombinant proteins are often targeted to the periplasm by an N-terminal signal peptide that is removed once it passes through the Sec-translocon in the cytoplasmic membrane. Despite their conserved targeting function, signal peptides can impact recombinant protein production yields in the periplasm, as can the production rate. Here, we present a combined screen involving different signal peptides and varying production rates that enabled the identification of more optimal conditions for periplasmic production of recombinant proteins with disulfide bonds. The data was generated from two targets, a single chain antibody fragment (BL1) and human growth hormone (hGH), with four different signal peptides and a titratable rhamnose promoter-based system that enables the tuning of protein production rates. Across the screen conditions, the yields for both targets significantly varied, and the optimal signal peptide and rhamnose concentration differed for each protein. Under the optimal conditions, the periplasmic BL1 and hGH were properly folded and active. Our study underpins the importance of combinatorial screening approaches for addressing the requirements associated with the production of a recombinant protein in the periplasm.

## Introduction

The bacterium *Escherichia coli* is one of the most widely used hosts to produce recombinant proteins (Rosano and Ceccarelli, 2014). While the majority of recombinant proteins are produced in the cytoplasm of *E. coli*, it is common to produce recombinant proteins that contain disulfide bonds in the periplasm. By targeting these proteins to the periplasm, it is possible to take advantage of the oxidizing environment and the resident disulfide bond formation (Dsb)-system to facilitate the proper disulfide bonds (Bardwell et al., 1991; Baneyx and Mujacic, 2004; Landeta et al., 2018). Although using the periplasm is more ideal for disulfide bond containing recombinant proteins, the bottlenecks associated with the targeting across the cytoplasmic membrane can substantially limit periplasmic yields (Baneyx and Mujacic, 2004; De Geyter et al., 2016).

To reach the periplasm, recombinant proteins are most often equipped with an N-terminal signal peptide that guides it to the Sec-translocon, which is a protein-conducting channel in the cytoplasmic membrane (Denks et al., 2014; Crane and Randall, 2017; Tsirigotaki et al., 2017). The targeting to the Sec-translocon can occur either co-translationally *via* the SRP-pathway or post-translationally in a chaperone-dependent or -independent manner (Kim et al., 2000; Tsirigotaki et al., 2017). Currently, it is not clear how the particular signal peptide, the mature part of the secretory protein, or even the mRNA affect the mode of targeting to the Sec-translocon (Gouridis et al., 2009; Chatzi et al., 2017; Tsirigotaki et al., 2018). Independent of the targeting pathway, proteins directed to the Sec-translocon are ultimately translocated across the cytoplasmic membrane in an unfolded conformation (Arkowitz et al., 1993), and the signal peptide is removed by leader peptidase (Paetzel, 2014; Tsirigotaki et al., 2017). Due to the requirements for translocation, the folding reaction is limited until these proteins reach the periplasm where the Dsb-system can mediate disulfide bond formation and various catalysts can guide the folding process (Inaba, 2009; Landeta et al., 2018). Different signal peptides from both targeting pathways as well as engineered signal peptides have been used for the production of recombinant proteins in the periplasm (Low et al., 2013; Zhou et al., 2016; Freudl, 2018; Selas Castineiras et al., 2018; Zhang et al., 2018). Thus far, it has not been possible to predict which signal peptide is optimal for the production of a particular recombinant protein in the periplasm.

Usually, the gene encoding a recombinant protein is expressed at the highest level possible (Wagner et al., 2008). For recombinant proteins that carry a signal peptide this can lead to saturation of the Sec-translocon capacity which can negatively affect biomass formation and protein production yields (Schlegel et al., 2013; Hjelm et al., 2017; Baumgarten et al., 2018). To overcome this bottleneck, our laboratory developed a rhamnose promoter-based system, which enables the precise regulation of protein production rates (Hjelm et al., 2017). Recently, we have shown that when a rhamnose promoter is used to govern the expression of the gene encoding a recombinant protein in a RhaT-mediated rhamnose transport and rhamnose catabolism deficient double mutant background, rhamnose promoter-based protein production rates can be regulated in a rhamnose concentration-dependent manner. This setup has successfully been used to avoid saturation of the Sec-translocon capacity during the production of a secretory recombinant protein, which leads to enhanced periplasmic protein production levels.

Numerous studies have shown that signal peptides and secretory protein production rates can independently influence the yields of periplasmic proteins, but these two aspects have not been examined in combination. The aim of this study was to examine the effects on periplasmic protein production when combining these two aspects. Hence, we produced two recombinant proteins containing disulfide bonds, the single chain variable fragment (scFv) BL1 and human growth hormone (hGH), using four signal peptides at different protein production rates. To vary the protein production rates aforementioned rhamnose promoter-based setup was used. For both target proteins a setup for enhanced production was identified using the signal peptide and production rate-based combinatorial screening approach.

## Materials and Methods

### Construction of *E. coli* W3110*ΔrhaΔlac*

To delete the *rha* operon and the *lac* operon in W3110 (obtained from the American Type Culture Collection) the Red-swap-method was used (Datsenko and Wanner, 2000). In short, kanamycin cassettes with regions homologous to the 5′ and 3′ flanking regions of the *rha* operon and the *lac* operon were generated by PCR using the pKD13 plasmid as a template and the primer pairs listed in Supplementary Table S1. The template was digested with *Dpn*I (NEB cutsmart) and the molecular weight of the PCR products were verified using an agarose gel and isolated using the Thermo Fisher Scientific gel extraction kit. To generate the W3110*rha*::Km^*R*^ and W3110*lac*::Km^*R*^ strains, the purified PCR products were electroporated into W3110 cells harboring pKD46 that had been cultured at 30°C in standard Lysogeny broth (LB) medium (Difco) containing 0.2% arabinose. Kanamycin-resistant clones (kan: 50 μg/ml final concentration) were then screened for the proper kanamycin cassette insertion by PCR using the primer pairs listed in Supplementary Table S1. Using P1-mediated generalized transduction, the region of interest of the strains exposed to the lambda Red system were transferred to cells that had not been exposed to the lambda Red system (Thomason et al., 2007). Upon successful transduction of the genetic region of interest, cells were transformed with pCP20 to remove the kanamycin cassette from the genome using FLP-recombinase (Datsenko and Wanner, 2000) and removal of the cassette was verified by PCR/sequencing. Finally, the cells were cured from pCP20 by a prolonged cultivation at 37°C. To generate the W3110*ΔrhaΔlac* strain, which is referred to in the text as *E. coliΔrha*, the *rha* operon in W3110 was deleted and then the *lac* operon was deleted from the resulting strain. It is of note that the removal of the *lac* operon prevents any secondary effects on the model recombinant protein scFv BL1 that could occur from binding to its substrate *E. coli* β-galactosidase (Schlegel et al., 2013).

### Construction of Expression Vectors

To create the expression vectors for the signal peptide-BL1-His_6_ constructs, i.e., DsbA_sp_BL1His_6_, Hbp_sp_BL1His_6_, OmpA_sp_BL1His_6_, and PhoA_sp_BL1His_6_ the gene encoding BL1 was amplified with forward primers containing the signal peptide coding sequence with an *Eco*RI site as an overhang (Supplementary Table S1) and a reverse primer that contained the His_6_-tag coding sequence with a *Hin*dIII site overhang (Schlegel et al., 2013). The PCR products encoding BL1 with a C-terminal His_6_-tag and the four different N-terminal signal peptides were then cloned into a pRha67Km^*R*^-derived vector (Giacalone et al., 2006; Hjelm et al., 2017). To create the expression vectors for the signal peptide-hGH-His_6_ fusion constructs, i.e., DsbA_sp_hGHHis_6_, Hbp_sp_hGHHis_6_, OmpA_sp_hGHHis_6_, and PhoA_sp_hGHHis_6_ and, the BL1 plasmids described above were used as templates for Gibson assembly where the BL1 coding region was replaced by a gene encoding hGH that is optimized for expression in *E. coli* (Browning et al., 2017). This resulted in four plasmids where hGH was N-terminally fused to a different signal peptide and C-terminally fused to a His_6_-tag. The primers for the Gibson assembly are listed in Supplementary Table S1. All plasmids were verified by sequencing. Plasmid sequences will be provided upon request.

### Protein Production Experiments

For all protein production experiments the *E. coli* strain W3110*ΔrhaΔlac* (referred to as *E. coliΔrha* in the main text) was used. Cells were transformed with the expression vectors described in the previous section or an empty expression vector that served as a control. Protein production screening was done in a standard 24-well plate format (culture volume 4 ml). Cells were grown aerobically at 30°C and 200 rpm (New Brunswick Innova 42R shaker with an orbit diameter of 1.9 cm), in LB medium supplemented with 50 μg/ml kanamycin. To precultures, 0.2% glucose was added to prevent background expression of the genes encoding the target proteins. Growth was monitored by measuring the A_600_ with a UV-1300 spectrophotometer (Shimadzu). At an A_600_ of ∼0.5 target gene expression was induced by the addition of rhamnose. For scFv BL1 production screening the following rhamnose concentrations were used: 0, 50, 100, 250, 500, and 5000 μM. For hGH production screening the following rhamnose concentrations were used: 0, 10, 50, 100, 250, and 5000 μM. Cells were harvested at the indicated time points for further analysis. Standard deviations shown in figures of culturing experiments are based on at least three independent biological replicates. Cultures for the isolation of hGH and BL1 were done in 2.5 L shake flasks (Tunair^TM^) containing 1 L of LB medium.

### SDS-PAGE, Coomassie Staining and Immuno-Blotting

To monitor the production of the scFv BL1 and hGH, proteins in whole-cell lysates were separated by sodium dodecyl sulfate-polyacrylamide gel electrophoresis (SDS-PAGE) followed by either Coomassie blue staining or immuno-blotting. scFv BL1 was analyzed on Tris Glycine 8–16% (Invitrogen) gels and hGH was analyzed using Tricine 16% (Invitrogen) gels. Lysates equivalent to 0.04 A_600_ units were loaded onto gels for Coomassie staining and lysates equivalent to 0.02 A_600_ units were loaded onto gels that were analyzed by immuno-blotting. For volumetric comparisons, cell pellets derived from 1 ml of culture were solubilized in 300 μl of sample buffer and 8 μl was loaded for Coomassie blue staining and 4 μl for immuno-blotting. In the blotting experiments, secretory/secreted BL1 and hGH were detected based on their C-terminal His_6_-tag using HisProbe-HRP Conjugate (Pierce) or the AlexaFluor 647 conjugated anti-HIS antibody (Invitrogen). The HisProbe-HRP Conjugate was used for mere detection and the AlexaFluor 647 conjugated anti-HIS antibody for quantification. The Western Bright Sirius kit (Advansta) was used to visualize immuno-blots stained with the HisProbe-HRP Conjugate. Chemiluminescence and fluorescence signals were detected using an Azure c600 imaging system (Azure Biosystems).

### Protein Isolation

Cells producing the scFv BL1 and hGH from 1 L cultures were sedimented at 6000 × *g* for 20 min, 4°C. The supernatant was discarded and the cell pellets were stored at −80°C. The cell pellets were thawed on ice and subsequently lysed in 25 ml buffer A [50 mM Tris–HCl pH 7.4 (for BL1) or pH 8 (for hGH), 0.1% Triton X-100, 1 mg/ml lysozyme, 1× Roche protease inhibitor cocktail, 1 mM phenylmethylsulfonyl fluoride (PMSF)] using the EmusiFlex C3 homogenizer (Avestin) in a cold room. The cell lysate was sedimented (10.000 × *g* for 20 min, 4°C) and the supernatant was transferred into a 70 Ti ultracentrifugation tube and sedimented again (100.000 × *g* for 1 h, 4°C). The high-spin supernatant was transferred to a 50 ml falcon tube, adjusted to a final concentration of 10 mM imidazole and 1 ml of a Ni-NTA agarose beads slurry was added that was pre-equilibrated with buffer B [1× phosphate buffered saline (PBS) pH 7.4, 150 mM NaCl, 0.1% Triton X-100, 10% Glycero]) containing 10 mM imidazole. The mixture was incubated in a cold room overnight with gentle tumbling and then transferred into a 10 ml polyprep chromatographic column. The column was washed with 20 column volumes (CV) of buffer B supplemented with 20 mM imidazole and the BL1 or hGH was eluted with buffer B supplemented with 300 mM imidazole. The eluted protein samples were pooled and loaded onto a HiLoad 16/60 Superdex 200 size exclusion chromatography (SEC) column mounted on an ÄKTAprime plus (GE Healthcare) that was pre-equilibrated with buffer C (50 mM Tris–HCl pH 7.4 (for BL1) or pH 8 (for hGH), 150 mM NaCl, 10% Glycerol). The column was run at a flow-rate of 1 ml/min, the protein was monitored at a wavelength of 280 nm, and the fractions corresponding to the peak were collected and stored at −20°C. All purification steps were done in the cold room. Protein concentration and purity were determined with the BCA method (Pierce) and SDS-PAGE followed by Coomassie staining. Protein folding was monitored using reducing and non-reducing SDS-PAGE followed by Coomassie staining and immuno-blotting. For BL1 analysis 2 μg of protein was loaded for Coomassie staining and 0.5 μg of protein for immuno-blotting and for hGH analysis 2 μg of protein was loaded for Coomassie staining and 1 μg of protein for immuno-blotting.

### BL1 Activity Assay

The proper folding of the isolated BL1 was assayed by the recognition of its substrate, *E. coli* β-galactosidase, using a dot-blot assay (Schlegel et al., 2013). For the activity assay, 100 μl of a 1:2 serial dilution of β-galactosidase (100, 50, 25, 12.5, 6.25, 3.125, 1.563, and 0 μg/ml) was spotted directly onto a nitrocellulose membrane (Millipore) using a BIO-DOT device (Bio-Rad). As a negative control, equal amounts of bovine serum albumin (BSA) were spotted on the same membrane. The membranes were blocked with a solution of tris buffered saline containing 0.05% Tween 20 (TBS-T) and 5% milk for 1 h at room temperature (RT), washed three times for 15 min with TBS-T and incubated overnight at 4°C with 17 μg/ml of the isolated BL1 in buffer C. The bound BL1 was labeled with the HisProbe-HRP Conjugate and the membrane was developed using the Western Bright Sirius kit and an Azure c600 imaging system.

### Human Growth Hormone Activity Assay

The activity of the purified hGH protein was ascertained by its ability to promote the growth of prolactin-dependent Nb2 lymphoma cell line (Nb2-11 cells) as previously described with slight modifications (Sonoda and Sugimura, 2008; Kim et al., 2013). Nb2-11 cells (Sigma) were cultured in RPMI 1640 medium (Gibco/Thermo Fisher Scientific) containing 10% fetal bovine serum (FBS), 10% horse serum (Gibco/Thermo Fisher Scientific) and 1% penicillin-streptomycin (Gibco/Thermo Fisher Scientific) at 37°C in a humidified atmosphere containing 5% CO_2_. To begin the assay, the cells were sedimented (500 × *g* for 3 min), washed once in medium without FBS and re-suspended in medium without FBS. The cell density was then determined with a Countess II automated cell counter (Life Technologies), diluted to approximately 2 × 10^5^ cells/ml and 100 μl of the cell suspension was added to each well of a 96 well plate. Each well then received the following amounts of the purified hGH protein: 0, 0.1, 0.5, and 1 ng. After mixing the solution on a shaker at 100 rpm for 1 min, the cells were placed in the 37°C humidified incubator and the cell proliferation 48 h after the hGH addition was used to determine its activity. To measure the cell growth, 20 μl of CellTiter 96^®^ aqueous one solution reagent (Promega) was added to each well, the plate was placed back in the 37°C humidified incubator for 2.5 h and then the absorbance was measured at 490 nm using a SpectraMax M2e microplate reader (Molecular Devices).

### Quantification of the scFv BL1 and hGH Amounts Produced in the Periplasm

Cells from 1 ml of either a 4 ml or 1 L culture were sedimented and lysed using 0.3 ml of sample buffer. The amounts of periplasmic BL1 and hGH in the samples were determined by measuring the mature levels of the proteins by immuno-blotting as follows. For BL1 samples a Tris Glycine 8–16% gel was loaded with 3 μl and a two-fold dilution of the whole cell lysate followed by a two-fold dilution series of the isolated BL1 containing 2, 1, 0.5, 0.25, 0.1, and 0.05 μg of protein. The samples were then resolved, transferred to a PVDF membrane, and the BL1 was detected with a fluorescently labeled antibody that recognizes the His_6_-tag. Images were acquired using an Azure c600 system. The band intensities were quantified with ImageJ and the values from the standard curve were used to determine the amount of BL1 in the two samples. The amount of hGH was determined similarly with the exception that a Bis Tris 4–12% gel was loaded with 10 μl and a two-fold dilution of the whole cell lysate and the dilution series of isolated hGH had the following amounts of protein: 3, 2, 1, 0.5, 0.25, 0.1, and 0.05 μg.

## Results and Discussion

### Combinatorial Signal Peptide and Production Rate Screening Approach

**FIGURE 1:**
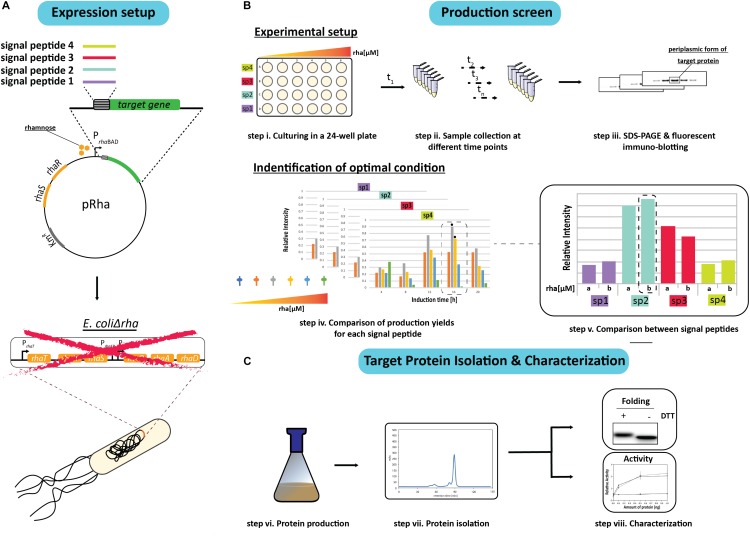
Setup of the signal peptide and production rate-based combinatorial screening approach to enhance periplasmic protein production yields in *E. coli*. **(A)** The gene encoding the protein to be produced in the periplasm is fused to the genetic information encoding different signal peptides. The genetic fusions are subsequently cloned in a rhamnose promoter-based expression vector (pRha). The expression vectors harboring the genes encoding the different signal peptide target protein fusions are subsequently transformed into an *E. coli* strain lacking the rhamnose operon (*E. coliΔrha)*. **(B)** Protein production screening is done in standard 24-well plates (step i). Expression of the genes encoding the different signal peptide target protein fusions is induced using varying amounts of rhamnose. Protein production in the periplasm is monitored over time (t_1_, t_*n*_) (step ii) by separating proteins present in equal volumes of culture by SDS-PAGE followed by immuno-blotting using a fluorescently labeled antibody recognizing the target protein (step iii). This enables to make for each of the used signal peptides a relative comparison of the protein production yields in the periplasm (step iv). The two highest periplasmic protein production yields are indicated with a black hexagon. For each signal peptide the two setups leading to the highest production yields are compared directly (step v). **(C)** This exercise is used to pick the condition used for scaling up target protein production (step vi) and the subsequent isolation of the target protein (step vii). The isolated protein is analyzed using SDS-PAGE in the presence and absence of the reductant DTT followed by Coomassie staining and immuno-blotting as well as a protein specific activity assay (step viii).

In an effort to identify the ideal condition for producing an oxidized recombinant protein in the *E. coli* periplasm, a combinatorial screen was set up using a panel of different signal peptides and a titratable rhamnose promoter system for controlling protein production rates. To initiate the screen, the genes encoding the target proteins with a C-terminal His_6_-tag were fused to sequences encoding for the signal peptides from the *E. coli* proteins DsbA, OmpA, PhoA, and the hemoglobin protease (Hbp) autotransporter, and inserted into the rhamnose promoter-based expression vector pRha (Figure 1A). These signal peptides were chosen because they are (i) from *E. coli* proteins, (ii) are commonly used for periplasmic targeting of recombinant proteins, and/or (iii) the corresponding mature proteins are reported to be rather abundant in the *E. coli* cell (Kendall et al., 1986; Schierle et al., 2003; Sijbrandi et al., 2003; Schlegel et al., 2013; Schmidt et al., 2016; Hjelm et al., 2017; Baumgarten et al., 2018) (see for more information Supplementary Table S2). The plasmids were then transformed into an *E. coli* strain (W3110*ΔrhaΔlac*) that enables tuning of the target production rates by varying the rhamnose concentration. The rhamnose tunability of the strain, herein referred to as *E.coliΔrha*, was achieved by deleting the whole *rha* operon, which includes the removal of the genes involved in rhamnose transport (*rhaT*) and catabolism (*rhaB*) that were previously shown to create a rhamnose titratable protein production strain (Hjelm et al., 2017).

Due to sample number limitations, the initial screen for each target protein with a particular signal peptide was analyzed independently using 24-well plates and a range of rhamnose concentrations to induce the target gene expression (Figure 1B). Following the induction, the cells from equal culture volumes were harvested at different time intervals and the periplasmic protein production was monitored by measuring the mature protein amounts using immuno-blots stained with fluorescently labeled antibodies (Figure 1B). The resulting data was then used to identify the best conditions for producing the target protein with each of the four signal peptides. To validate that the observed improvements in periplasmic production were not associated with the culture volume analysis, the production levels in equal cell amounts was also examined using Coomassie staining and immuno-blotting.

Based on the results from the initial screen, the two best conditions for each target protein and signal peptide combination were compared directly to determine the rhamnose concentration and signal peptide that provided the highest periplasmic yields (Figure 1B). The most effective signal peptide and rhamnose concentration was then tested using large scale cultures where the target proteins were isolated, quantified and characterized for disulfide bond formation and activity (Francis et al., 2002; Figure 1C). Two targets were used throughout this study, the single chain antibody fragment BL1 (scFv BL1), which recognizes the *E. coli* β-galactosidase (Schlegel et al., 2013), and human growth hormone (hGH), which stimulates cell proliferation (Kim et al., 2013). Both targets were chosen because they contain two disulfide bonds in their native state and a C-terminal His_6_-tag was incorporated for isolation and detection.

### ScFv BL1 Production Screen

**FIGURE 2:**
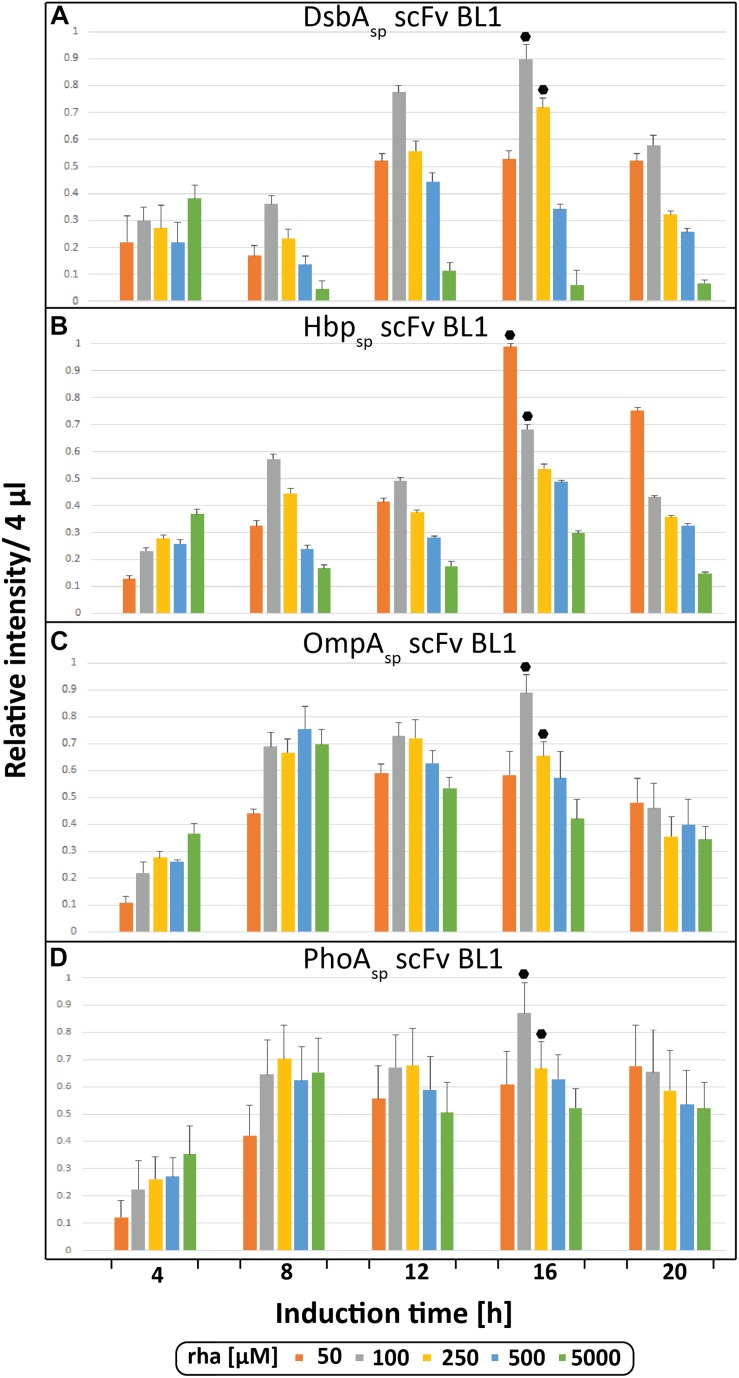
Periplasmic production of the scFv BL1 using different signal peptides and inducer concentrations monitored volumetrically over time. Production of the scFv BL1 N-terminally fused to the DsbA **(A)**, the Hbp **(B)**, the OmpA **(C),** and the PhoA **(D)** signal peptide, in *E. coliΔrha* using varying amounts of rhamnose, was monitored over time (4, 8, 12, 16, and 20 h after induction of target gene expression). Proteins present in equal amounts of culture volume were separated by SDS-PAGE followed by immuno-blotting using a fluorescently labeled antibody recognizing the His_6_-tag fused to the C-terminus of BL1 (see section “Materials and Methods”). Fluorescent signals representing BL1 (i.e., the processed/periplasmic form of the protein) were quantified using ImageJ and peak intensities were used for comparison. To enable the comparison of BL1 production between different time-points for each signal peptide, standard curves were made using peak intensities from serial dilutions of the optimal condition of each signal peptide. For each signal peptide used the two highest periplasmic BL1 production yields are indicated with a black hexagon.

During the initial screen the temporal production of the scFv BL1 in *E. coliΔrha* was examined using the four different signal peptides (DsbA_sp_, Hbp_sp_, OmpA_sp_, and PhoA_sp_) and a range of rhamnose concentrations (Figure 2). The BL1 amounts, which were determined for equal culture volumes by SDS-PAGE immune-blots (Figure 1B), showed that for all signal peptides the highest BL1 yields were obtained 16 h post-induction with rhamnose. The production of BL1 with each signal peptide was then examined at 16 h post-induction using equal amounts of biomass (Supplementary Figures S1, S2). While the optimal rhamnose concentration slightly changed for some signal peptides in the equal biomass analysis based on Coomassie staining and immuno-blotting against the C-terminal His_6_, the preference for lower rhamnose concentrations was confirmed.

**FIGURE 3:**
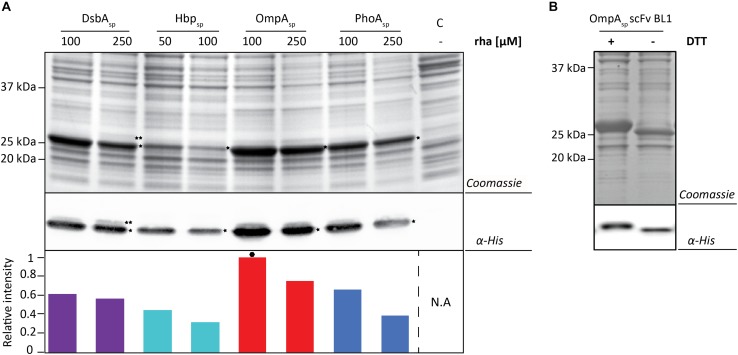
Identifying the optimal setup for the periplasmic production of the scFv BL1. **(A)** For each signal peptide used the two setups leading to the highest periplasmic production yields of BL1 were compared using SDS-PAGE followed by Coomassie staining and immuno-blotting using a fluorescently labeled antibody recognizing the His_6_-tag at the C-terminus of BL1. Importantly, equal amounts of culture volume were loaded per lane since this enables to directly compare the amount of BL1 produced in the periplasm volumetrically. Relative intensities of the fluorescent signals are shown below the blot. The highest periplasmic BL1 production yield is indicated with a black hexagon (see also Supplementary Figure S3). The precursor form, i.e., BL1 with the signal peptide still attached, is indicated with ^∗∗^ and the mature form, i.e., BL1 without signal peptide, is indicated with ^*^. **(B)** The use of the OmpA-BL1 fusion resulted in the highest production of BL1 in the periplasm. To assess the folding of the BL1 produced in the periplasm cells were solubilized in sample buffer with and without the reductant DTT prior to SDS-PAGE followed by Coomassie staining and immuno-blotting using HisProbe.

Next, the BL1 production yields for each signal peptide at the two best rhamnose conditions were directly compared using equal culture volumes (Figure 3A). The results from this volumetric analysis revealed that for BL1 the highest periplasmic production yields were achieved with the OmpA signal peptide and 100 μM rhamnose. To monitor if BL1 produced with the OmpA signal peptide had formed disulfide bonds in the periplasm, the whole cell lysates were separated by SDS-PAGE in the presence and the absence of the reductant DTT followed by Coomassie staining and immuno-blotting (Figure 3B). Indicative that BL1 is properly folded with two intramolecular disulfide bonds, BL1 migrated more slowly when it was reduced by DTT.

**FIGURE 4:**
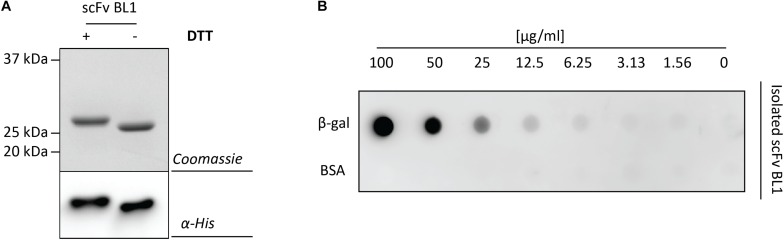
Characterization of the isolated scFv BL1. For the isolation of BL1, the OmpA signal peptide and 100 μM of rhamnose for the induction of target gene expression were used (see Figure 3A). BL1 was isolated using a combination of IMAC and SEC. **(A)** The isolated BL1 was incubated in sample buffer with and without the reductant DTT prior to SDS-PAGE followed by Coomassie staining and immuno-blotting using HisProbe. **(B)** A nitrocellulose membrane containing increasing amounts of β-galactosidase as well as increasing amounts of BSA (control) was incubated with the isolated BL1. Binding of BL1 to the β-galactosidase spotted on the nitrocellulose membrane was detected using HisProbe recognizing the His_6_-tag at the C-terminus of BL1.

To more thoroughly examine the periplasmic BL1 that was produced with the most optimal signal peptide and rhamnose concentration, BL1 was produced in a larger scale culture and subsequently isolated using immobilized-metal affinity chromatography (IMAC) followed by size exclusion chromatography (SEC). The isolated BL1 showed the expected migration shift in the presence of DTT (Figure 4A). To confirm that the isolated BL1 is indeed properly folded, we examined its ability to bind β-galactosidase (Figure 4B; Schlegel et al., 2013). For this analysis, β-galactosidase and BSA were spotted in decreasing concentrations on a nitrocellulose membrane. The membrane was then incubated with the isolated BL1 and the bound BL1 was detected using HisProbe (see section “Materials and Methods”). As expected, the BL1 produced under the optimal conditions was capable of binding to its substrate and showed no affinity for the BSA negative control.

Together, these results show that by screening different signal peptides and protein production rates for the scFv BL1 we were able to identify conditions capable of producing functional BL1 in the periplasm at around 200 μg per ml of culture (Supplementary Figure S4).

### hGH Production Screen

**FIGURE 5:**
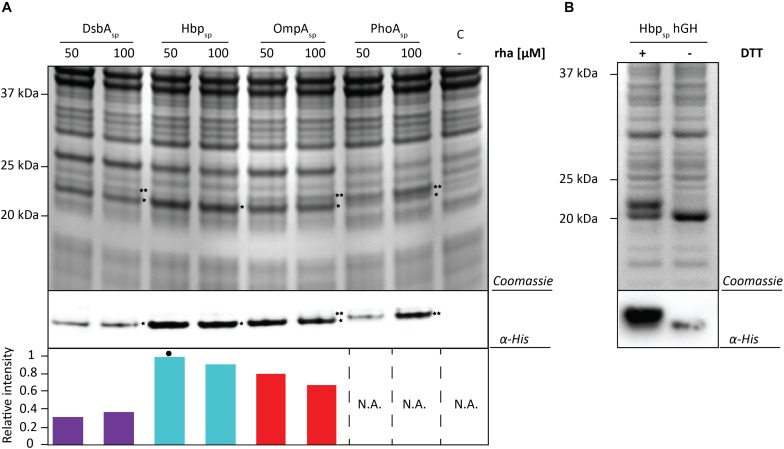
Identifying the optimal setup for the periplasmic production of hGH. **(A)** For each signal peptide used, the two setups leading to the highest periplasmic production yields of hGH were compared by SDS-PAGE followed by Coomassie staining and immuno-blotting using a fluorescently labeled antibody recognizing the His_6_-tag at the C-terminus of hGH Importantly, equal amounts of culture volume were loaded per lane since this enables to compare the amount of hGH produced in the periplasm volumetrically. Relative intensities of the fluorescent signals are shown below the blot. The highest periplasmic hGH production yield is indicated with a black hexagon (see also Supplementary Figure S7). The precursor form, i.e., hGH with the signal peptide still attached, is indicated with ^∗∗^ and the mature form, i.e., hGH without signal peptide, is indicated with ^*^. **(B)** Whole cell lysates derived from cells producing hGH fused to the Hbp signal peptide at 50 μM of rhamnose isolated 16 h after the addition of rhamnose, were analyzed by means of SDS-PAGE followed by Coomassie staining and immuno-blotting (see section “Materials and Methods”). To assess the folding of the hGH produced in the periplasm cells were dissolved in sample buffer with and without the reductant DTT prior to SDS-PAGE (see also Supplementary Figure S8).

To test if the target can have an influence on the yields obtained with each signal peptide and the different production rates, the screen was repeated using hGH and the two best production conditions were identified for each signal peptide (Figure 5A and Supplementary Figures S5, S6). Similar to the results using BL1, the highest hGH yields were observed at 16 h post-induction with rhamnose for all of the signal peptides. For hGH the use of the Hbp signal peptide and 50 μM rhamnose resulted in the highest production of hGH in the periplasm. To monitor if hGH produced with the Hbp signal peptide had formed disulfide bonds in the periplasm whole cell lysates were analyzed using SDS-PAGE in the presence and absence of DTT (Figure 5B). As expected, the mature hGH showed slower mobility in the presence of DTT, indicating that it likely folds correctly and obtains its two intramolecular disulfide bonds. Thus, both signal peptide and rhamnose concentration leading to the highest production of hGH in the periplasm are different from the ones observed for BL1.

**FIGURE 6:**
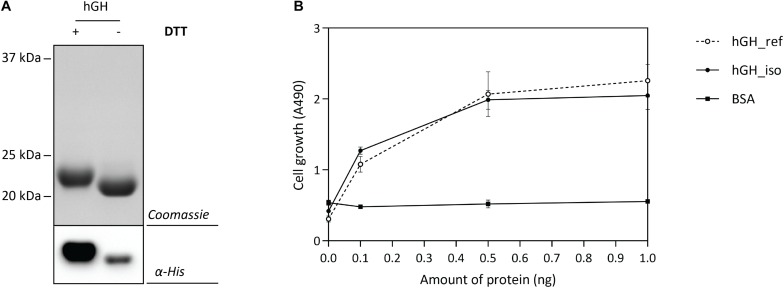
Characterization of isolated hGH. For the isolation of hGH, the Hbp signal peptide and 50 μM of rhamnose for the induction of target gene expression were used (see Figure 5). hGH was isolated using a combination of IMAC and SEC. **(A)** To assess the folding of the isolated hGH, it was solubilized in sample buffer with and without the reductant DTT prior to SDS-PAGE followed by Coomassie staining and immuno-blotting using HisProbe (see also Supplementary Figure S8). **(B)** The activity of the isolated hGH was assessed by monitoring its ability to promote growth of an Nb2-11 cell line. Resuspended Nb2-11 cells in medium without FBS were incubated with various amounts of isolated hGH (filled circle), hGH reference (empty circle), and BSA (filled square) in a 96-well plate and cultured at 37°C in a humidified, 5% CO_2_ atmosphere for 48 h. Cell proliferation was determined colorimetrically with MTT reagent. hGH reference and BSA were used as positive and negative control, respectively. All experiments were done in triplicate and standard deviations are depicted by error bars.

The optimal Hbp-signal peptide-based hGH production setup was scaled up and a combination of IMAC and SEC was used to isolate hGH. Isolated hGH was analyzed by means of SDS-PAGE in the presence and the absence of the reductant DTT followed by Coomassie staining and immuno-blotting (Figure 6A). The shift of isolated hGH toward a lower molecular weight upon omitting DTT is an indication that the hGH produced is properly folded. Next, we examined the ability of the isolated hGH to promote the growth of prolactin-dependent Nb2 lymphoma cell line (Nb2-11 cells) using commercially available hGH as a reference and BSA as a negative control (Figure 6B). Using this set-up, we showed that hGH produced under the optimal conditions was capable of promoting the growth of prolactin-dependent Nb2 lymphoma cell line equally well as commercially purchased hGH.

Taken together, here we show for hGH that screening different signal peptides and protein production rates leads to the identification of a setup that enables to produce functional hGH in the periplasm at around 30 μg per ml of culture.

## Concluding Remarks

Here, to enhance the production of the scFv BL1 and hGH in the periplasm we used a combinatorial screening approach using four different signal peptides and a rhamnose promoter-based setup enabling to precisely set protein production rates. Main finding is that the signal peptide and rhamnose concentration that provide the highest production yields in the periplasm are different for the two target proteins that were tested. Although the induction times for the optimal conditions are the same for both targets, this may be a mere coincidence and needs to be confirmed using additional targets before this parameter can be fixed.

Previously, it has been shown that the mature part of *E. coli* secretory proteins can contain information that is important for their secretion and that a signal peptide can affect the folding/targeting of a protein destined for the periplasm (Liu et al., 1989; Rusch et al., 2002; Gouridis et al., 2009; Singh et al., 2013; Chatzi et al., 2017; Tsirigotaki et al., 2018). How these observations translate to the production of heterologous proteins in the periplasm of *E. coli* is still *terra incognita*. However, the effects observed for BL1 and hGH production using different signal peptides strongly suggest that signal peptide - target protein combinations can have a profound effect on the production of a protein in the periplasm. Striking example is that the use of the PhoA signal peptide leads to good - although not the best – periplasmic production of BL1, whereas hardly any hGH is produced in the periplasm when the PhoA signal peptide is used. Recently, for PhoA it has been shown that its efficient translocation depends on so-called mature domain targeting signals (Chatzi et al., 2017). Interestingly, using the MatureP predictor, which was trained using the *E. coli* proteome, such a mature domain targeting signal in BL1 could be identified, whereas in hGH such a signal could not be identified (Orfanoudaki et al., 2017). This may explain why the PhoA signal peptide only promotes the efficient export of BL1. These observations raise the question if it would be possible to develop predictors which can streamline screening for the enhanced production of recombinant proteins in the periplasm.

In conclusion, our study shows that combinatorial screening of different signal peptides and protein production rates opens up a new avenue to enhance protein production yields in the periplasm of *E. coli*.

## Data Availability

The raw data supporting the conclusion of this manuscript will be made available by the authors, without undue reservation, to any qualified researcher.

## Author Contributions

AK, HA-K, RD, and J-WG conceived and designed the experiments. AK, HA-K, TH, AM, and KD conducted the experiments. AK, HA-K, TH, AM, KD, RD, and J-WG analyzed the data. AK, HA-K, KMD, SS, KR, RD, and J-WG wrote the manuscript.

## Conflict of Interest Statement

The authors declare that the research was conducted in the absence of any commercial or financial relationships that could be construed as a potential conflict of interest.

## References

[B1] Arkowitz R. A. Joly J. C. Wickner W. (1993). Translocation can drive the unfolding of a preprotein domain. *EMBO J.* 12 243–253. 10.1002/j.1460-2075.1993.tb05650.x 8428582 PMC413198

[B2] Baneyx F. Mujacic M. (2004). Recombinant protein folding and misfolding in *Escherichia coli*. *Nat. Biotechnol.* 22 1399–1408. 10.1038/nbt1029 15529165

[B3] Bardwell J. C. Mcgovern K. Beckwith J. (1991). Identification of a protein required for disulfide bond formation *in vivo*. *Cell* 67 581–589. 10.1016/0092-8674(91)90532-4 1934062

[B4] Baumgarten T. Ytterberg A. J. Zubarev R. A. De Gier J. W. (2018). Optimizing recombinant protein production in the *Escherichia coli* periplasm alleviates stress. *Appl. Environ. Microbiol.* 84 e00270-18. 10.1128/AEM.00270-18 29654183 PMC5981079

[B5] Browning D. F. Richards K. L. Peswani A. R. Roobol J. Busby S. J. W. Robinson C. (2017). *Escherichia coli* “TatExpress” strains super-secrete human growth hormone into the bacterial periplasm by the Tat pathway. *Biotechnol. Bioeng.* 114 2828–2836. 10.1002/bit.26434 28842980 PMC5698719

[B6] Chatzi K. E. Sardis M. F. Tsirigotaki A. Koukaki M. Sostaric N. Konijnenberg A. (2017). Preprotein mature domains contain translocase targeting signals that are essential for secretion. *J. Cell Biol.* 216 1357–1369. 10.1083/jcb.201609022 28404644 PMC5412566

[B7] Crane J. M. Randall L. L. (2017). The sec system: protein export in *Escherichia coli*. *EcoSal Plus* 7 1–44. 10.1128/ecosalplus.ESP-0002-2017 PMC5807066 29165233

[B8] Datsenko K. A. Wanner B. L. (2000). One-step inactivation of chromosomal genes in *Escherichia coli* K-12 using PCR products. *Proc. Natl. Acad Sci. U.S.A.* 97 6640–6645. 10.1073/pnas.120163297 10829079 PMC18686

[B9] De Geyter J. Tsirigotaki A. Orfanoudaki G. Zorzini V. Economou A. Karamanou S. (2016). Protein folding in the cell envelope of *Escherichia coli*. *Nat. Microbiol.* 1:16107. 10.1038/nmicrobiol.2016.107 27573113

[B10] Denks K. Vogt A. Sachelaru I. Petriman N. A. Kudva R. Koch H. G. (2014). The sec translocon mediated protein transport in prokaryotes and eukaryotes. *Mol. Membr. Biol.* 31 58–84. 10.3109/09687688.2014.907455 24762201

[B11] Francis E. Daniels R. Hebert D. N. (2002). Analysis of protein folding and oxidation in the endoplasmic reticulum. *Curr. Protoc. Cell Biol.* 14 15.6.1–15.6.29. 10.1002/0471143030.cb1506s14 18228398

[B12] Freudl R. (2018). Signal peptides for recombinant protein secretion in bacterial expression systems. *Microb. Cell Fact.* 17:52. 10.1186/s12934-018-0901-3 29598818 PMC5875014

[B13] Giacalone M. J. Gentile A. M. Lovitt B. T. Berkley N. L. Gunderson C. W. Surber M. W. (2006). Toxic protein expression in *Escherichia coli* using a rhamnose-based tightly regulated and tunable promoter system. *Biotechniques* 40 355–364. 10.2144/000112112 16568824

[B14] Gouridis G. Karamanou S. Gelis I. Kalodimos C. G. Economou A. (2009). Signal peptides are allosteric activators of the protein translocase. *Nature* 462 363–367. 10.1038/nature08559 19924216 PMC2823582

[B15] Hjelm A. Karyolaimos A. Zhang Z. Rujas E. Vikstrom D. Slotboom D. J. (2017). Tailoring *Escherichia coli* for the l-rhamnose PBAD promoter-based production of membrane and secretory proteins. *ACS Synth Biol.* 6 985–994. 10.1021/acssynbio.6b00321 28226208

[B16] Inaba K. (2009). Disulfide bond formation system in *Escherichia coli*. *J. Biochem.* 146 591–597. 10.1093/jb/mvp102 19567379

[B17] Kendall D. A. Bock S. C. Kaiser E. T. (1986). Idealization of the hydrophobic segment of the alkaline phosphatase signal peptide. *Nature* 321 706–708. 10.1038/321706a0 3520341

[B18] Kim J. Luirink J. Kendall D. A. (2000). SecB dependence of an exported protein is a continuum influenced by the characteristics of the signal peptide or early mature region. *J. Bacteriol.* 182 4108–4112. 10.1128/jb.182.14.4108-4112.2000 10869093 PMC94600

[B19] Kim M. J. Park H. S. Seo K. H. Yang H. J. Kim S. K. Choi J. H. (2013). Complete solubilization and purification of recombinant human growth hormone produced in *Escherichia coli*. *PLoS One* 8:e56168. 10.1371/journal.pone.0056168 23409149 PMC3567055

[B20] Landeta C. Boyd D. Beckwith J. (2018). Disulfide bond formation in prokaryotes. *Nat. Microbiol.* 3 270–280. 10.1038/s41564-017-0106-2 29463925

[B21] Liu G. Topping T. B. Randall L. L. (1989). Physiological role during export for the retardation of folding by the leader peptide of maltose-binding protein. *Proc. Natl. Acad Sci. U.S.A.* 86 9213–9217. 10.1073/pnas.86.23.9213 2687876 PMC298464

[B22] Low K. O. Muhammad Mahadi N. Md Illias R. (2013). Optimisation of signal peptide for recombinant protein secretion in bacterial hosts. *Appl. Microbiol. Biotechnol.* 97 3811–3826. 10.1007/s00253-013-4831-z 23529680

[B23] Orfanoudaki G. Markaki M. Chatzi K. Tsamardinos I. Economou A. (2017). MatureP: prediction of secreted proteins with exclusive information from their mature regions. *Sci. Rep.* 7:3263. 10.1038/s41598-017-03557-4 28607462 PMC5468347

[B24] Paetzel M. (2014). Structure and mechanism of *Escherichia coli* type I signal peptidase. *Biochim. Biophys. Acta* 1843 1497–1508. 10.1016/j.bbamcr.2013.12.003 24333859

[B25] Rosano G. L. Ceccarelli E. A. (2014). Recombinant protein expression in *Escherichia coli*: advances and challenges. *Front. Microbiol.* 5:172. 10.3389/fmicb.2014.00172 24860555 PMC4029002

[B26] Rusch S. L. Mascolo C. L. Kebir M. O. Kendall D. A. (2002). Juxtaposition of signal-peptide charge and core region hydrophobicity is critical for functional signal peptides. *Arch. Microbiol.* 178 306–310. 10.1007/s00203-002-0453-z 12209265

[B27] Schierle C. F. Berkmen M. Huber D. Kumamoto C. Boyd D. Beckwith J. (2003). The DsbA signal sequence directs efficient, cotranslational export of passenger proteins to the *Escherichia coli* periplasm via the signal recognition particle pathway. *J. Bacteriol.* 185 5706–5713. 10.1128/jb.185.19.5706-5713.2003 13129941 PMC193964

[B28] Schlegel S. Rujas E. Ytterberg A. J. Zubarev R. A. Luirink J. De Gier J. W. (2013). Optimizing heterologous protein production in the periplasm of *E. coli* by regulating gene expression levels. *Microb. Cell Fact* 12:24. 10.1186/1475-2859-12-24 23497240 PMC3605120

[B29] Schmidt A. Kochanowski K. Vedelaar S. Ahrne E. Volkmer B. Callipo L. (2016). The quantitative and condition-dependent *Escherichia coli* proteome. *Nat. Biotechnol.* 34 104–110. 10.1038/nbt.3418 26641532 PMC4888949

[B30] Selas Castineiras T. Williams S. G. Hitchcock A. Cole J. A. Smith D. C. Overton T. W. (2018). Development of a generic beta-lactamase screening system for improved signal peptides for periplasmic targeting of recombinant proteins in *Escherichia coli*. *Sci. Rep.* 8:6986. 10.1038/s41598-018-25192-3 29725125 PMC5934370

[B31] Sijbrandi R. Urbanus M. L. Ten Hagen-Jongman C. M. Bernstein H. D. Oudega B. Otto B. R. (2003). Signal recognition particle (SRP)-mediated targeting and Sec-dependent translocation of an extracellular *Escherichia coli* protein. *J. Biol. Chem.* 278 4654–4659. 10.1074/jbc.M211630200 12466262

[B32] Singh P. Sharma L. Kulothungan S. R. Adkar B. V. Prajapati R. S. Ali P. S. (2013). Effect of signal peptide on stability and folding of *Escherichia coli* thioredoxin. *PLoS One* 8:e63442. 10.1371/journal.pone.0063442 23667620 PMC3646739

[B33] Sonoda H. Sugimura A. (2008). Improved solubilization of recombinant human growth hormone inclusion body produced in *Escherichia coli*. *Biosci. Biotechnol. Biochem.* 72 2675–2680. 10.1271/bbb.80332 18838803

[B34] Thomason L. C. Costantino N. Court D. L. (2007). *E. coli* genome manipulation by P1 transduction. *Curr. Protoc. Mol. Biol*. 79 1.17.1–1.17.8. 10.1002/0471142727.mb0117s79 18265391

[B35] Tsirigotaki A. Chatzi K. E. Koukaki M. De Geyter J. Portaliou A. G. Orfanoudaki G. (2018). Long-lived folding intermediates predominate the targeting-competent secretome. *Structure* 26 695.e5–707.e5. 10.1016/j.str.2018.03.006 29606594

[B36] Tsirigotaki A. De Geyter J. Sostaric N. Economou A. Karamanou S. (2017). Protein export through the bacterial Sec pathway. *Nat. Rev. Microbiol.* 15 21–36. 10.1038/nrmicro.2016.161 27890920

[B37] Wagner S. Klepsch M. M. Schlegel S. Appel A. Draheim R. Tarry M. (2008). Tuning *Escherichia coli* for membrane protein overexpression. *Proc. Natl. Acad Sci. U.S.A.* 105 14371–14376. 10.1073/pnas.0804090105 18796603 PMC2567230

[B38] Zhang W. Lu J. Zhang S. Liu L. Pang X. Lv J. (2018). Development an effective system to expression recombinant protein in E. coli via comparison and optimization of signal peptides: expression of *Pseudomonas fluorescens* BJ-10 thermostable lipase as case study. *Microb. Cell Fact* 17:50. 10.1186/s12934-018-0894-y 29592803 PMC5872382

[B39] Zhou Y. Liu P. Gan Y. Sandoval W. Katakam A. K. Reichelt M. (2016). Enhancing full-length antibody production by signal peptide engineering. *Microb. Cell Fact* 15:47. 10.1186/s12934-016-0445-3 26935575 PMC4776426

