# Supplementary material for: Enhancing Recombinant Protein Yields in the E. coli Periplasm by Combining Signal Peptide and Production Rate Screening
Source: Front Microbiol. 2019 Jul 23;10:1511. doi: 10.3389/fmicb.2019.01511 (PMC6664373; doi:10.3389/fmicb.2019.01511)
Supplement: Supplementary file 1 [file Data_Sheet_1.PDF]

Supplementary Information

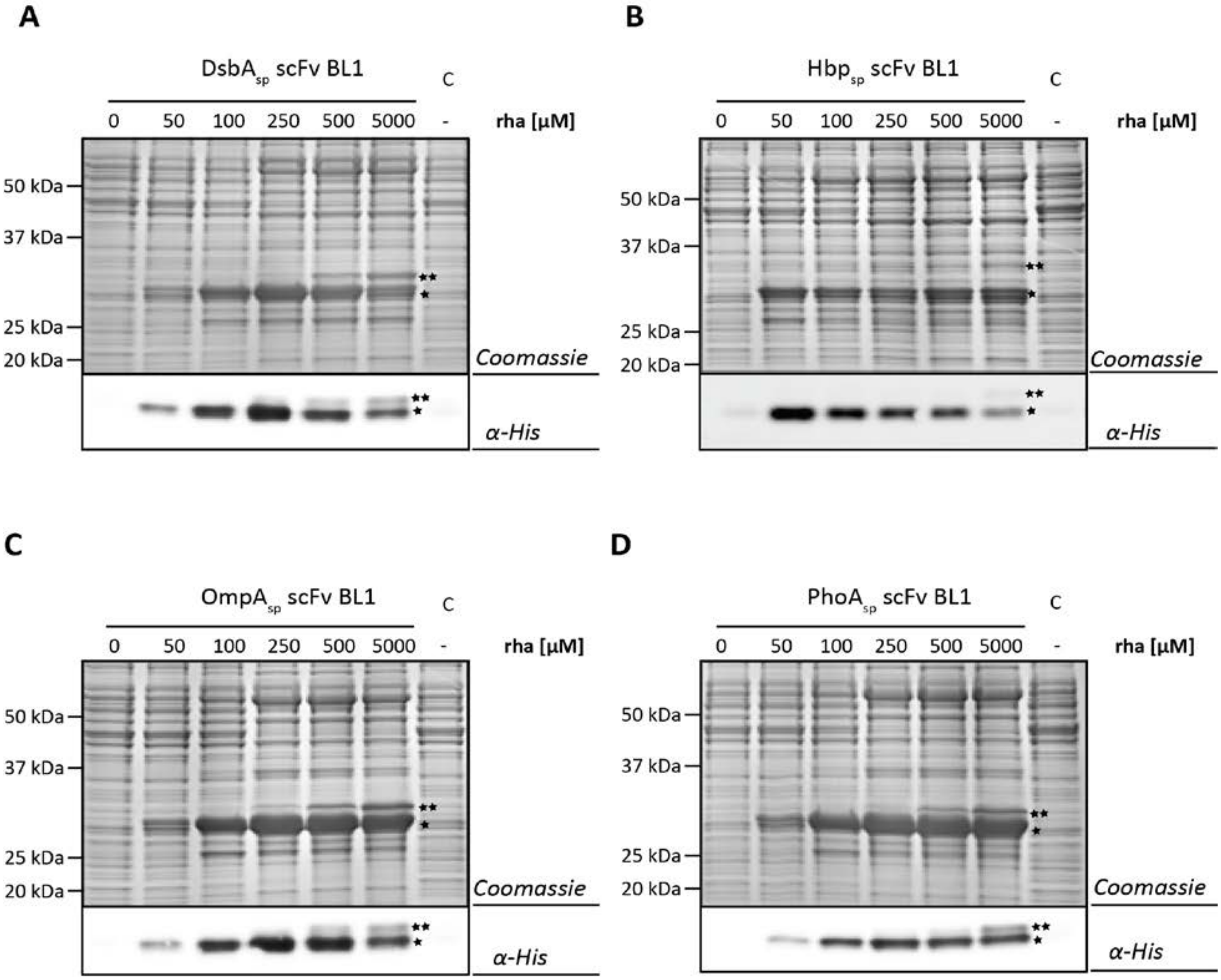

Figure S1. ScFv BL1 production screen. The scFv BL1 N-terminally fused to the DsbA (A), the Hbp (B), the OmpA (C) and the PhoA (D) signal peptide was produced in *E. coliΔrha* at varying concentrations of rhamnose. Cells were harvested 16 h after induction of target gene expression with rhamnose since at this time-point the highest volumetric production yields were obtained. The protein content of equal amounts of cells was analyzed by SDS-PAGE followed by Coomassie staining (see Materials and Methods). For the biomass formed in cultures producing the scFv BL1 16 h after gene expression see Figure S2. The precursor form, *i.e.*, BL1 with the signal peptide still attached, is indicated with ** and the mature form, *i.e.*, BL1 without signal peptide, is indicated with *. Target protein production was also monitored by means of immuno-blotting using a fluorescently labelled antibody recognizing the His_6-tag at the C-terminus of BL1 (see Materials and Methods).

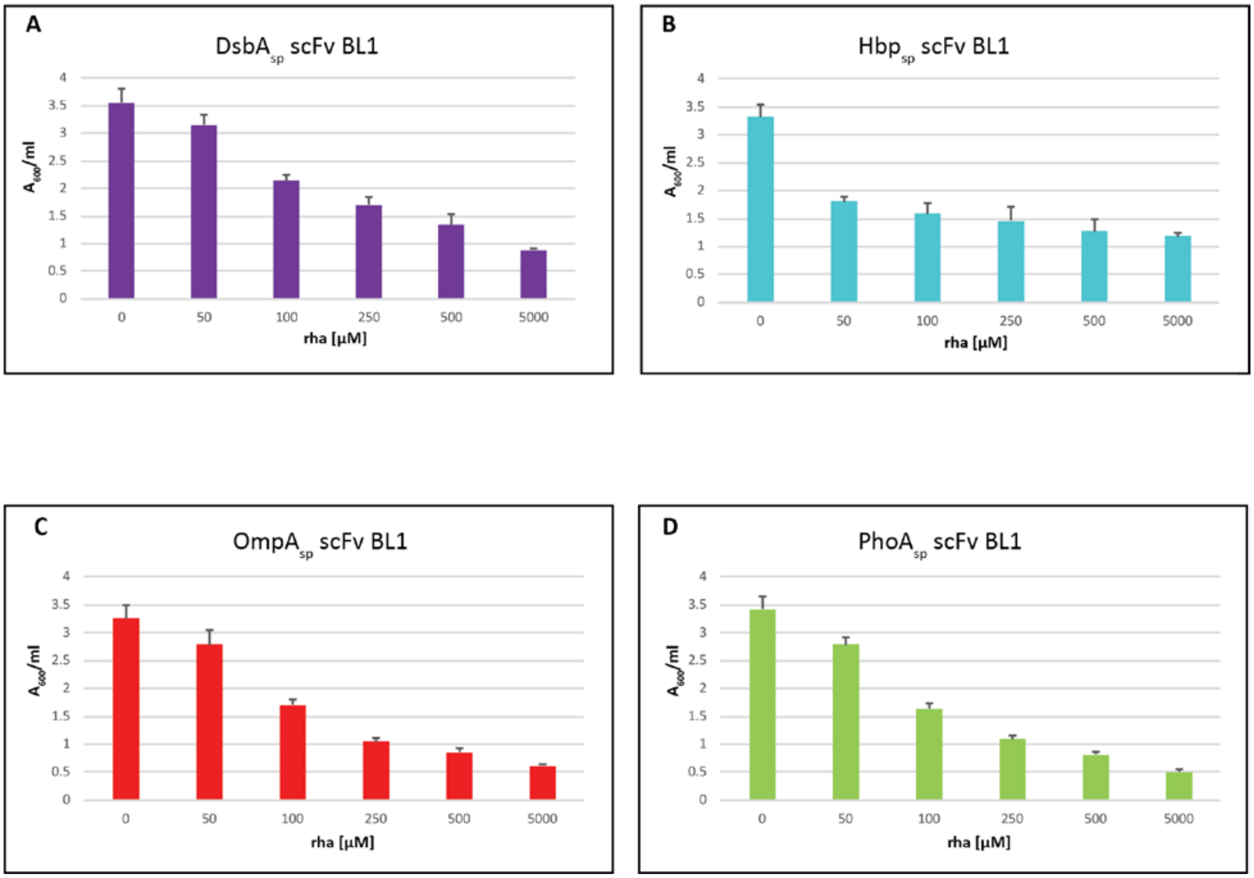

Figure S2. Biomass formation in cultures producing the scFv BL1 16 h after induction of target gene expression. Biomass formation in cultures producing BL1 fused to the DsbA (A), the Hbp (B), the OmpA (C) and the PhoA (D) signal peptide at different rhamnose concentrations was monitored by measuring A_{600} values per ml of culture (see Materials and Methods). Data shown are based on three independent biological replicates.

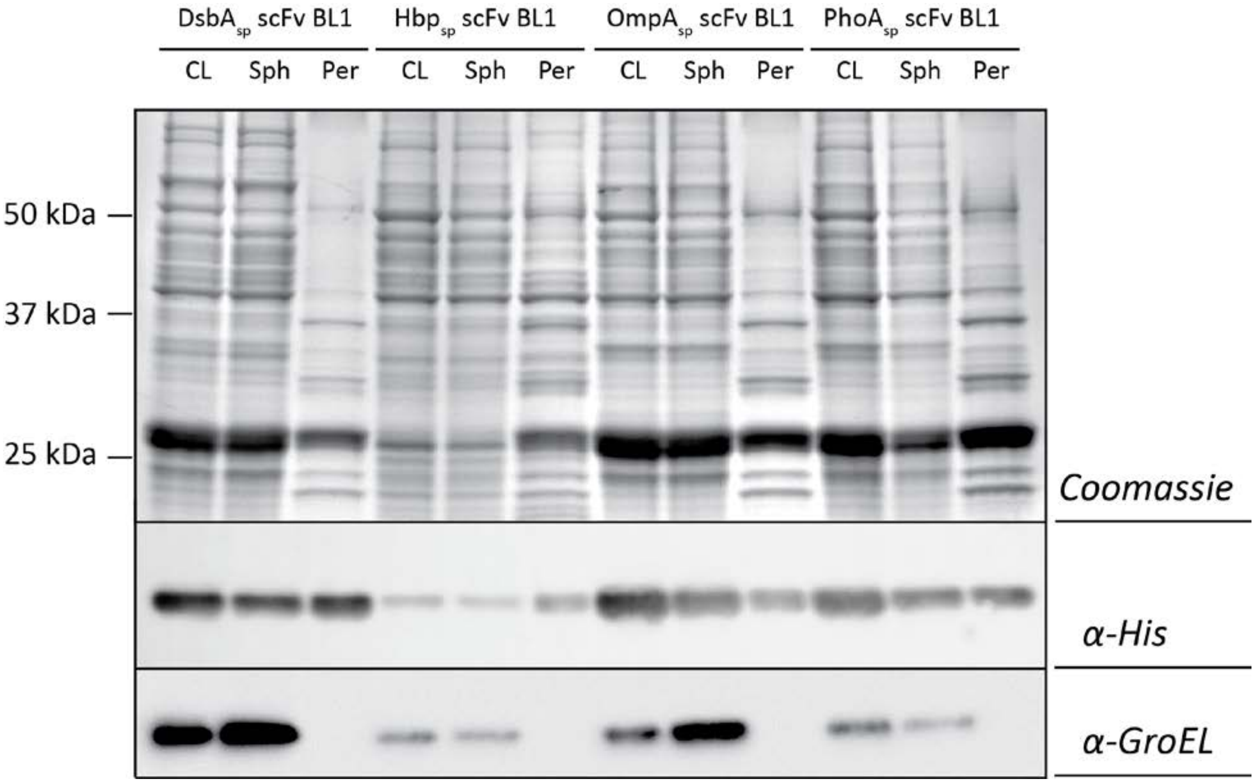

Figure S3. Subcellular fractionation of *E. coliΔrha* cells producing secretory scFv BL1. Secretory BL1 was produced in *E. coliΔrha* in the presence of 100 µM rhamnose for DsbA$_{sp}$, OmpA$_{sp}$ and PhoA$_{sp}$ and 50 µM for the Hbp$_{sp}$ as described in the 'Materials and Methods' section. Spheroplasts were generated by incubating cells in TSE buffer (200 mM Tris–HCl, pH 8.0, 500 mM sucrose, 1 mM EDTA) on ice/at 4°C and subsequently the periplasmic fraction was isolated essentially as described previously (Quan et al., 2013). Protein concentrations of the different fractions were determined using the BCA assay (Pierce) as described in the 'Materials and Methods' section. Samples corresponding to 10 µg of total protein were analyzed using SDS-PAGE followed by immuno-blotting. BL1 was detected using a fluorescently labelled α-His antibody as described in the 'Materials and Methods' section. The chaperone GroEL was used as cytoplasmic marker and detected using a polyclonal anti-serum against GroEL raised in a rabbit. The GroEL immuno-blot was developed using HRP conjugated secondary antibodies raised against rabbit antibodies (see the 'Materials and Methods' section). **CL:** Whole cell lysate, **Sph:** Spheroplast fraction and **Per:** Periplasmic fraction.

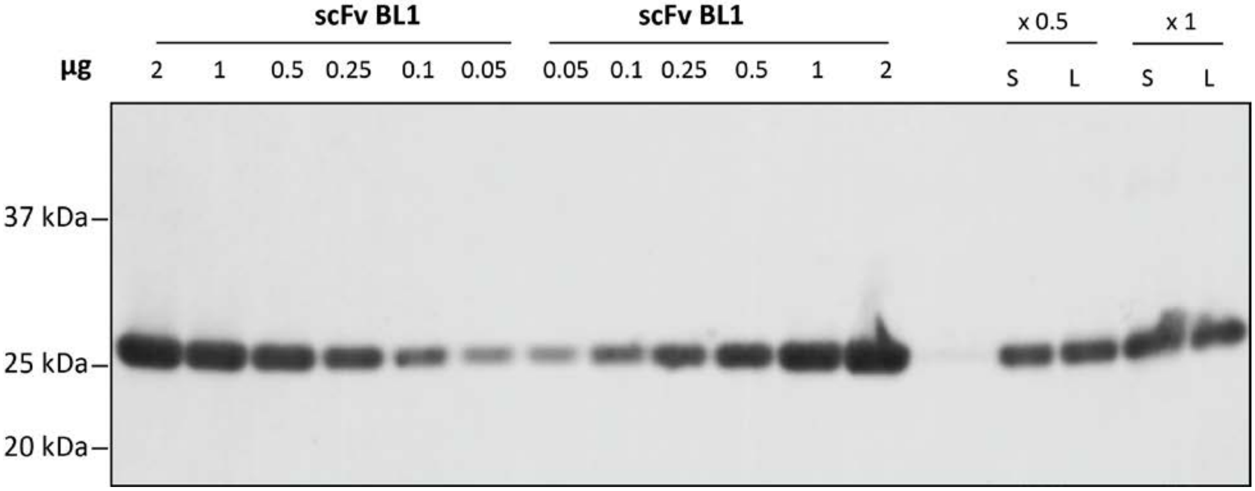

Figure S4. **Quantification of the amount of the scFv BL1 produced in the periplasm using the OmpA signal peptide.** Proteins present in equal amounts of culture volume were separated by SDS-PAGE followed by immuno-blotting using a fluorescently labelled antibody recognizing the His_6-tag fused to the C-terminus of BL1 (see Materials and Methods). Cells from 1 ml of culture from a 4 ml culture (24 well plate) (Small) and a 1 L culture (Large) were harvested and subsequently resuspended in 1 ml of sample buffer. A two-fold dilution (0.5x) was prepared from the initial sample (1x) and 3 µl of both the dilution and the initial sample were loaded on the gel. Two standard curves were prepared to assist with computing the amount of periplasmic BL1 produced. The amounts of isolated BL1 loaded are 2, 1, 0.5, 0.25, 0.1 and 0.05 µg (see Materials and Methods). Fluorescent signals were quantified using ImageJ. For the 4 ml culture 182.97 ± 10.40 µg of BL1 per ml of culture was produced and for the 1 L culture 219.49 ± 8.45 µg of BL1 per ml of culture was produced. Standard deviations are based on two gels each containing two standard curves.

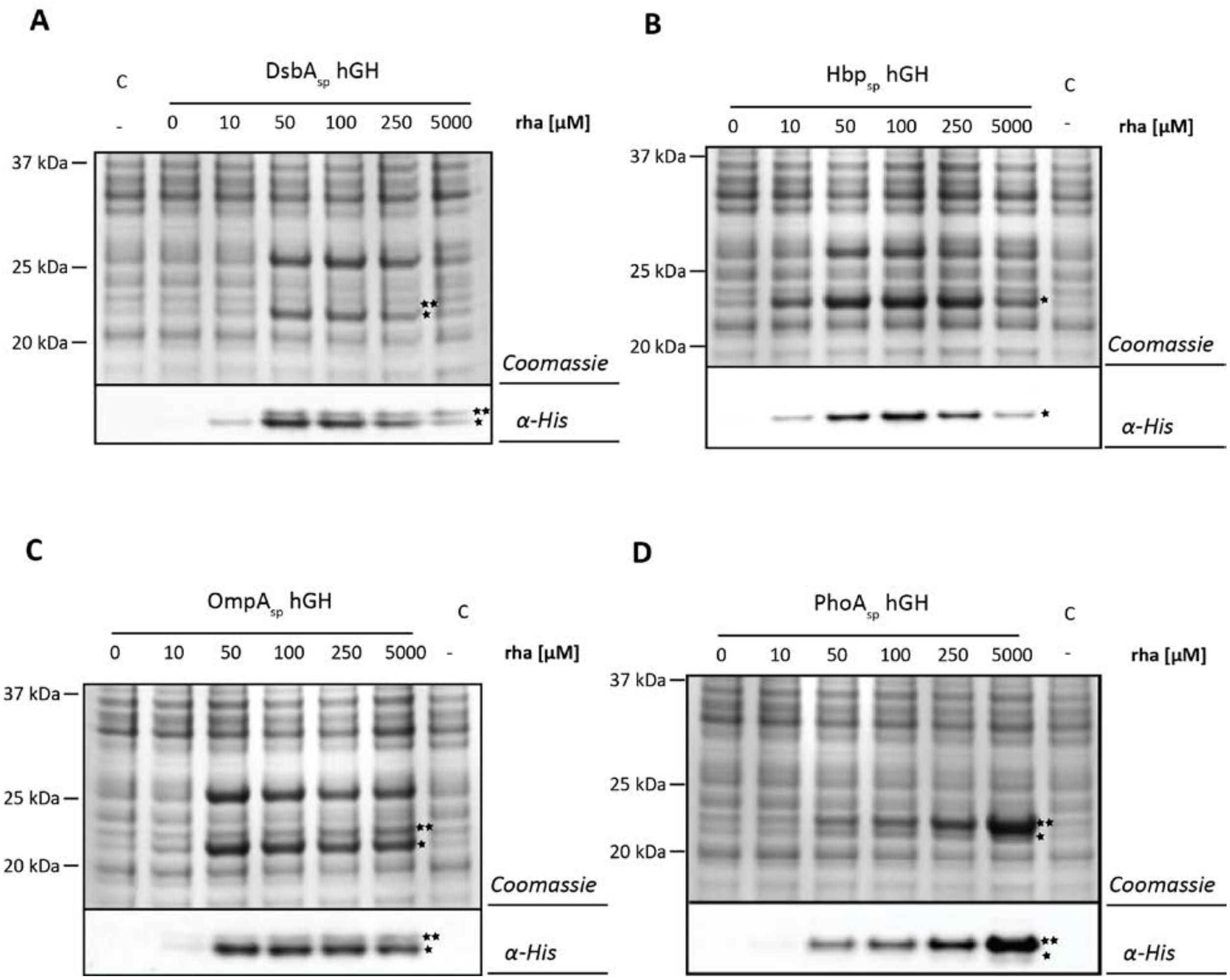

Figure S5. hGH production screen. hGH N-terminally fused to the DsbA (A), the Hbp (B), the OmpA (C) and the PhoA (D) signal peptide was produced in *E. coliΔrha* at varying concentrations of rhamnose. Cells were harvested 16 h after induction of target gene expression with rhamnose since at this time-point the highest volumetric production yields were obtained. The protein content of equal amounts of cells was analyzed by SDS-PAGE followed by Coomassie staining (see Materials and Methods). For the biomass formed in cultures producing hGH 16 h after gene expression see Figure S5. The precursor form, *i.e.*, hGH with the signal peptide still attached, is indicated with ** and the mature form, *i.e.*, hGH without signal peptide, is indicated with *. Target protein production was also monitored by means of immuno-blotting using a fluorescently labelled antibody recognizing the His_6-tag at the C-terminus of hGH (see Materials and Methods).

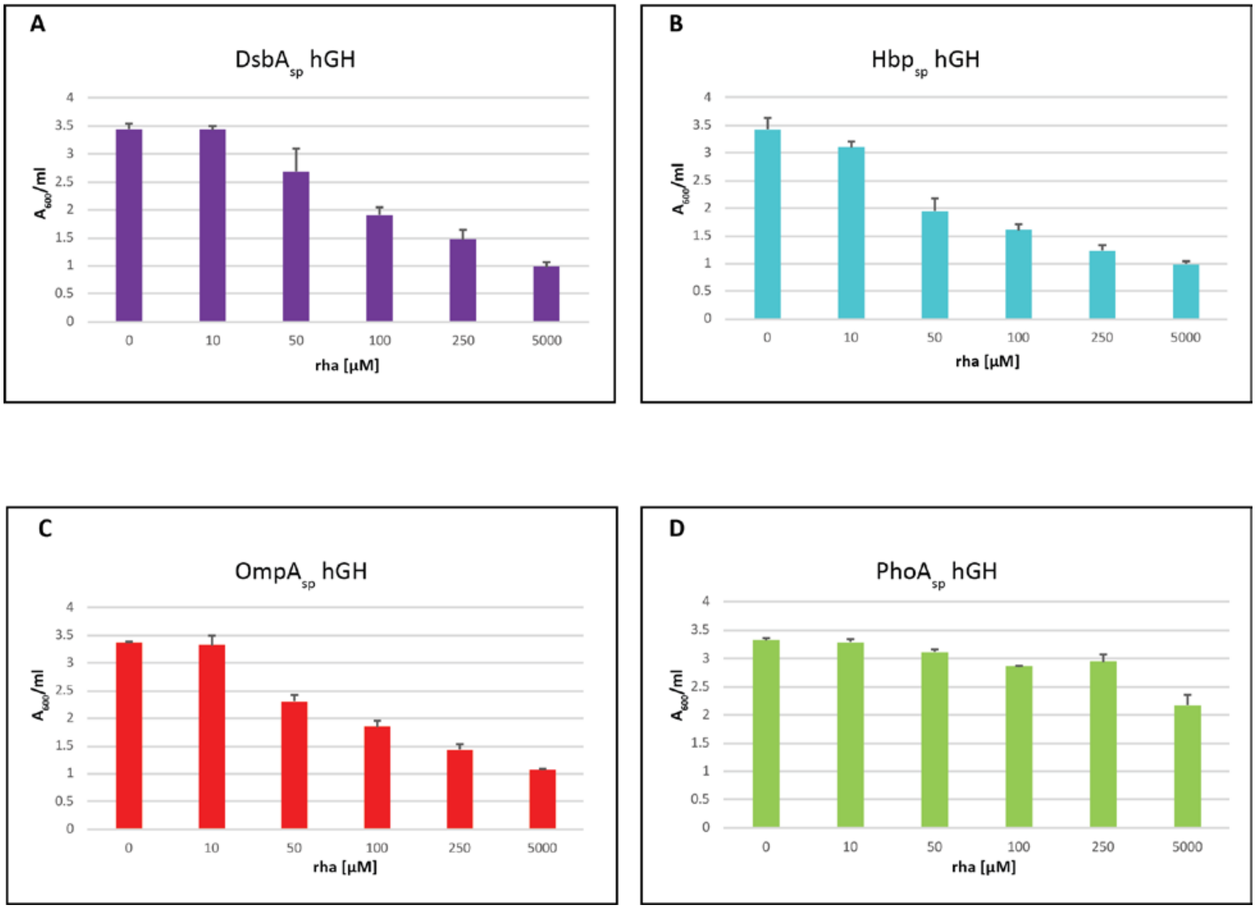

Figure S6. Biomass formation in cultures producing hGH after 16 h of induction. Biomass formation in cultures producing hGH fused to the DsbA (A), the Hbp (B), the OmpA (C) and the PhoA (D) signal peptide at different rhamnose concentrations was monitored by measuring A_{600} values per ml of culture (see Materials and Methods). Data shown are based on three independent biological replicates.

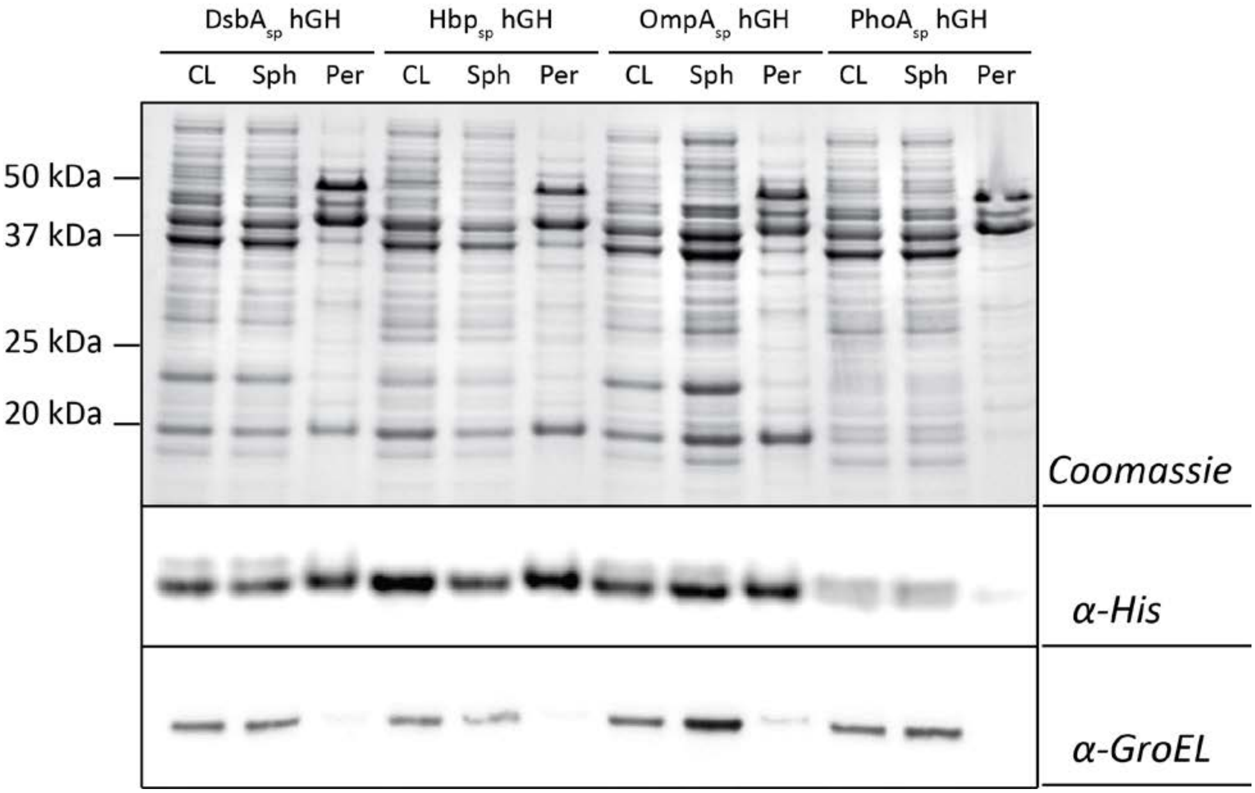

Figure S7. Subcellular fractionation of *E. coliΔrha* cells producing secretory hGH. Secretory hGH was produced in *E. coliΔrha* in the presence of 50 µM rhamnose as described in the 'Materials and Methods' section. Spheroplasts were generated by incubating cells in TSE buffer (200 mM Tris–HCl, pH 8.0, 500 mM sucrose, 1 mM EDTA) on ice/at 4°C and subsequently the periplasmic fraction was isolated essentially as described previously (Quan et al., 2013). Protein concentrations of the different fractions were determined using the BCA assay (Pierce) as described in the 'Materials and Methods' section. Samples corresponding to 10 µg of total protein were analyzed using SDS-PAGE followed by immuno-blotting. hGH was detected using a fluorescently labelled α-His antibody as described in the 'Materials and Methods' section. The chaperone GroEL was used as cytoplasmic marker and detected using a polyclonal anti-serum against GroEL raised in a rabbit. The GroEL immuno-blot was developed using HRP conjugated secondary antibodies raised against rabbit antibodies (see the 'Materials and Methods' section). **CL:** Whole cell lysate, **Sph:** Spheroplast fraction and **Per:** Periplasmic fraction.

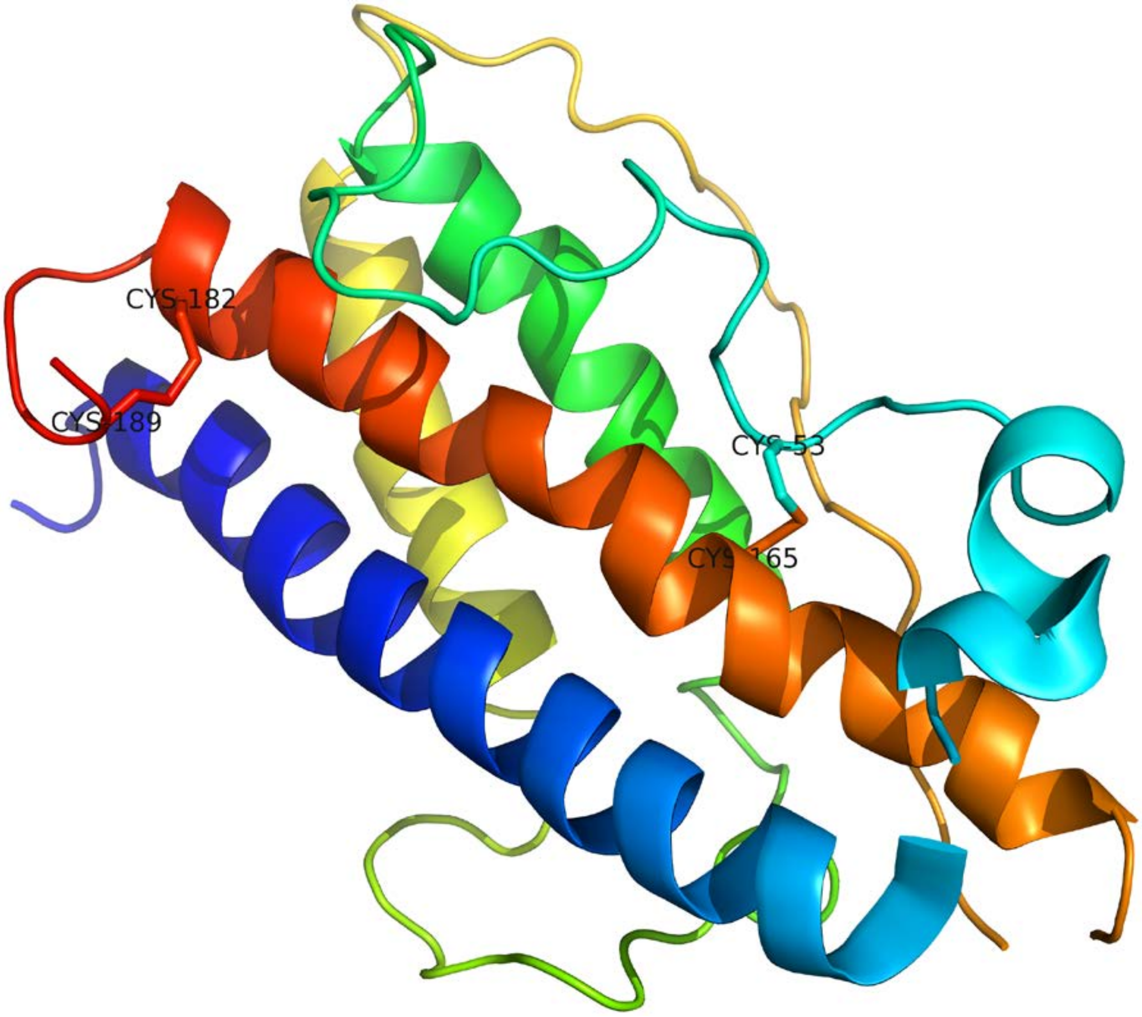

Figure S8. X-ray crystal structure of hGH. Cartoon representation of the x-ray crystal structure of hGH [PDB ID = 1HGU] (Chantalat et al., 1995) showing two disulfide bonds (CYS53-CYS165 and CYS182-CYS-189) in the protein. By reducing the disulfide bonds renders the C-terminal helix (in red) more flexible and extended, thereby making the His_6-tag at the C-terminus of hGH more accessible to the anti-His probe for protein detection in an immuno-blot. This explains why there is not a difference in Coomassie stained hGH-His_6 with and without the addition of the reductant DTT, but there is a difference when probing hGH-His_6 upon blotting with anti-His probe.

Table S1. Primers used in this study

dsbA-bl1-fw	CAGGAGGAATTCACCATGTTAAGATCCATGAAAAAG ATTTGGCTGGCGCTGGCTGGTTTAGTTTTAGCGTTTAG CGCATCGGCGATGGCCGAGGTGCAGCTGGTGG	Forward primer used to fuse the genetic information encoding the scFv BL1 to the genetic information encoding the DsbA signal peptide. The primer introduces an *Eco*RI site at the 5' end of the fusion.
hbp-bl1-fw	CAGGAGGAATTCACCATGAACAGAATTTATTCTCTTC GCTACAGCGCTGTGGCCCGGGGCTTTATTGCCGTATC TGAGTTTGCTAGGAAATGTGT TCATAAGTCTGTCAGACGTCTGTGTTTCCCGGTTTTAT TACTGATCCCGGTACTATTCTCTGCAGGAAGTCTTGCG ATGGCCGAGGTGCAGCTGGTG	Forward primer used to fuse the genetic information encoding the scFv BL1 to the genetic information encoding the Hbp signal peptide. The primer introduces an *Eco*RI site at the 5' end of the fusion..
ompA-bl1-fw	CAGGAGGAATTCACCATGAAAAAGACAGCTATCGCG ATTGCAGTGGCACTGGCTGGTTTCGCTACCGTAGCGC AGGCCATGGCCGAGGTGCAGCTGGTGG	Forward primer used to fuse the genetic information encoding the scFv BL1 to the genetic information encoding the OmpA signal peptide. The primer introduces an *Eco*RI site at the 5' end of the fusion..
phoA-bl1-fw	CAGGAGGAATTCACCGTGAAACAAAGCACTATTGCAC TGGCACTCTTACCGTTACTGTTTACCCCTGTGACAAAA GCCATGGCCGAGGTGCAGCTGGTGG	Forward primer used to fuse the genetic information encoding the scFv BL1 to the genetic information encoding the PhoA signal peptide. The primer introduces an *Eco*RI site at the 5' end of the fusion.
bl1-rv	ACAGCCAAGCTTTTATTAGTGATGATGATGATGATGA CTAGTT	Reverse primer to introduce a *Hin*dIII site to 3' end of the BL1 gene
dsbA-hgh-GA-A	GTTTTAGCGTTTAGCGCATCGGCGATGTTCCCAACCAT TCCCTTATCCAGG	Forward primer to amplify *hgh* and introduce an overlap homologous to pRha-dsbA$_{sp}$ for Gibson assembly.
dsbA-hgh-GA-B	GCCTGGATAAGGGAATGGTTGGGAACATCGCCGATGC GCTAAACGCTAAAAC	Reverse primer to amplify pRha-dsbA$_{sp}$ and introduce an overlap homologous to *hgh* for Gibson assembly.
hbp-hgh-GA-A	GTACTATTCTCTGCAGGAAGTCTTGCGATGTTCCCAAC CATTCCCTTATCCAGG	Forward primer to amplify *hgh* and introduce an overlap homologous to with pRha-hbp$_{sp}$ for Gibson assembly.

hbp-hgh-GA-B	GGATAAGGGAATGGTTGGGAACATCGCAAGACTTCCT GCAGAGAATAGTACC	Reverse primer to amplify pRha-hbp$_{sp}$ and introduce an overlap homologous to *hgh* for Gibson assembly.
ompA-hgh-GA-A	CTGGTTTCGCTACCGTAGCGCAGGCCATGTTCCCAAC CATTCCCTTATCCAGG	Forward primer to amplify *hgh* and introduce an overlap homologous to pRha-ompA$_{sp}$ for Gibson assembly.
ompA-hgh-GA-B	GCCTGGATAAGGGAATGGTTGGGAACATGGCCTGCGC TACGGTAGCGAAAC	Reverse primer to amplify pRha-ompA$_{sp}$ and introduce an overlap homologous to *hgh* for Gibson assembly.
phoA-hgh-GA-A	CTGTTTACCCCTGTGACAAAAGCCATGTTCCCAACCA TTCCCTTATCCAGG	Forward primer to amplify *hgh* and introduce an overlap homologous to pRha-phoA$_{sp}$ for Gibson assembly.
phoA-hgh-GA-B	GATAAGGGAATGGTTGGGAACATGGCTTTTGTCACAG GGGTAAACAGTAAC	Reverse primer to amplify pRha-phoA$_{sp}$ and introduce an overlap homologous to *hgh* for Gibson assembly.
hgh-GA-A	TCATCCGCCAAAACAGCCAAGCTTTTATTAGTGATGA TGATGATGATGGAAGCC	Forward primer to amplify *hgh* and introduce an overlap homologous to pRha for Gibson assembly.
pRha-GA-A	CCATCATCATCATCATCACTAATAAAAGCTTGGCTGTT TTGGCGGATGAGAG	Reverse primer to amplify pRha and introduce an overlap homologous to *hgh* for Gibson assembly.
rhaMCS-fw	CACGTTCATCTTTCCCTGGT	Forward primer to amplify the pRha multiple cloning site
rhaMCS-rv	CTGAAAATCTTCTCTCATCCGC	Reverse primer to amplify the pRha multiple cloning site
rha-FRT-fw	CAAATCGAAATTTAAAATATTGTGCCGGAGGCATCTC TGGCACATTGGGCAA ATTCCGGGGATCCGTCGACC	Forward primer to generate a Km^{R}-FRT cassette to delete the *rha* operon
rha-FRT-rv	CACTCATCTCGTCGGAGATGTGACGCGACGAAAAATG ATGAGGATAAGAAG TGTAGGCTGGAGCTGCTTCG	Reverse primer to generate a Km^{R}-FRT cassette to delete the *rha* operon
rha-up	GAGGCGATATGTCGGTAAACAATG	Forward primer to verify the *rha* operon deletion
rha-dw	GTTTGGTGTGGTGGATTTCCATGG	Reverse primer to verify the *rha* operon deletion
lac-FRT-fw	GCTGAACTTGTAGGCCTGATAAGCGCAGCGTATCAGG CAATTTTTATAAT ATTCCGGGGATCCGTCGACC	Forward primer to generate a Km^{R}-FRT cassette to delete the *lac* operon

lac-FRT-rv	GCGGTATGGCATGATAGCGCCCGGAAGAGAGTCAATT CAGGGTGGTGAATTGTAGGCTGGAGCTGCTTCG	Reverse primer to generate a Km^R-FRT cassette to delete the *lac* operon
lac-up	CTGGTTACCCGCTTTCTATATTGAG	Forward primer to verify the *lac* operon deletion
lac-dw	CAAAGAGGGTTACGGACAGAACTAC	Reverse primer to verify the *lac* operon deletion

Table S2. For the DsbA, Hbp, OmpA and PhoA signal peptides the following information is listed:

1. **Amino acid sequence** (extension n-region h-region c-region)
2. **Function and localization of the native protein, presence/absence of disulfide bonds** (Yes/No)
3. **Relevant information as to protein targeting**
4. **Examples of recombinant proteins produced using the signal peptide**

**DsbA**	1. MKKIWLALAGLVLAFSASA<br><br>2. DsbA is a periplasmic thiol:disulfide oxidoreductase which promotes protein disulfide bond formation in *E. coli* (Landeta et al., 2018). Disulfide bonds: Yes (depending on its state) (Bardwell et al., 1991).<br><br>3. The Beckwith laboratory has provided evidence that the DsbA signal peptide mediates the co-translational SRP-dependent targeting of proteins (Schierle et al., 2003). However, a ribosomal profiling study by the Bukau laboratory indicates that DsbA is actually discriminated by SRP (Schibich et al., 2016).<br><br>4. scFv BL1 (Schlegel et al., 2013), BJ-10 lipase (Zhang et al., 2018), hGH (Soares et al., 2003), MBP (Schierle et al., 2003), Human proinsulin (Winter et al., 2001), TrxA (Schierle et al., 2003), mAbs hu5D5 (Zhou et al., 2016), DARPins (Steiner et al., 2006)
**Hbp**	1. MNRIYSLRYSAVARGFIAVSEFARKCVHKSVRRLCFPVLLLIPVLFSAGSLA<br><br>2. The Hemoglobin protease (Hbp) is an autotransporter from *E. coli* EB1 (Otto et al., 1998). Disulfide bonds: No.<br><br>3. The Luirink laboratory has shown that the SRP mediates the co-translational targeting of Hbp and that SecB, which mediates mostly post-translational protein targeting, is not required for its targeting. However, in the absence of SRP, SecB can promote Hbp targeting (Sijbrandi et al., 2003).<br><br>4. Proteins/antigens displayed on the cell surface or secreted into the extracellular milieu as Hbp fusions: Calmodulin-1 (Jong et al., 2007), ESAT6 (Hjelm et al., 2015), ESAT6, Ag85B and Rv2660c (Jong et al., 2014), SpyTag/SnoopTag (van den Berg van Saparoea et al., 2018).

**OmpA**	1. MKKTAIAIAVALAGFATVAQA<br><br>2. OmpA is an *E. coli* outer membrane protein and believed to be an unspecific diffusion channel for small solutes (Sugawara and Nikaido, 1992).<br>Disulfide bonds: Yes (Negoda et al., 2010).<br><br>3. SecB is required for the efficient targeting of OmpA (Baars et al., 2006).<br><br>4. scFv BL1 (Baumgarten et al., 2018),Calcitonin peptide (Ray et al., 2002), Staphylokinase (Lee et al., 1998), Thermoalkaliphilic lipase (Rua et al., 1998), scFv F19, OS4, H398, TTX (Rippmann et al., 1998), Human and mouse Leptin (Guisez et al., 1998), Peptide:N-glycosidase F (Loo et al., 2002), Winter flounder antifreeze (Tong et al., 2000), Exoglucanase (Lam et al., 1997), hEGF (Sivakesava et al., 1999), Fungal ribotoxin α-sarcin (Rathore et al., 1997), FV Fragment-Tumor Necrosis Factor Alpha Fusion Protein (Yang et al., 1998), Mini-antibodies McPC603scFVDhlx (Kujau et al., 1998), Murein interleukin-2 (Robbens et al., 1995), TEM-β-lactamase (Wan and Baneyx, 1998), Cholera toxin B (Slos et al., 1994), tPA (Manosroi et al., 2001), hGH (Becker and Hsiung, 1986), Fab murine monoclonal antibody (Ellis et al., 2017).
**PhoA**	1. VKQSTIALALLPLLFTPVTKA<br><br>2. Alkaline phosphatase is a periplasmic, homodimeric enzyme that catalyses the hydrolysis and transphosphorylation of a wide variety of phosphate monoesters (Stec et al., 2000).<br>Disulfide bonds: Yes (Bradshaw et al., 1981) (Sone et al., 1997).<br><br>3. PhoA targeting has been reported to be both SecB-dependent (Kusukawa et al., 1989) and SecB-independent (Kumamoto and Beckwith, 1985), as well as partially SecB-dependent (Gannon et al., 1989; Crane and Randall, 2017). In addition, in a study from the Kendall laboratory it has been shown that the efficient targeting of PhoA may also be dependent on SRP (Kim et al., 2001). Recently, mature domain targeting signals in the mature domain of PhoA have been shown to play a role in the targeting efficiency of the pre-protein (Chatzi et al., 2017).<br><br>4. CRP (Tanaka et al., 2002), Cytochrome P450 (Kaderbhai et al., 2001), Glucagon (Wen et al., 2003), hEGF (Engler et al., 1988), hEGF (Zhang et al., 2007), Mouse Endostatin (Xu et al., 2002).

Abbreviations: CRP: Human C-reactive protein, **DARPins**: Designed ankyrin-repeat proteins, **Fab**: Antigen-binding fragment, **hEGF**: Human epidermal growth factor, **hGH**: Human growth hormone, **mAbs**: full-length monoclonal antibodies, **MBP**: Maltose binding protein, **scFv**: Single chain variable fragment, **SRP**: Signal recognition particle, **tPA**: Tissue Plasminogen Activator, **TrxA**: Thioredoxin 1

References

Baars, L., Ytterberg, A.J., Drew, D., Wagner, S., Thilo, C., Van Wijk, K.J., et al. (2006). Defining the role of the *Escherichia coli* chaperone SecB using comparative proteomics. J Biol Chem 281(15), 10024-10034. doi: 10.1074/jbc.M509929200.

Bardwell, J.C., Mcgovern, K., and Beckwith, J. (1991). Identification of a protein required for disulfide bond formation *in vivo*. Cell 67(3), 581-589.

Baumgarten, T., Ytterberg, A.J., Zubarev, R.A., and De Gier, J.W. (2018). Optimizing Recombinant Protein Production in the *Escherichia coli* Periplasm Alleviates Stress. Appl Environ Microbiol 84(12). doi: 10.1128/AEM.00270-18.

Becker, G.W., and Hsiung, H.M. (1986). Expression, secretion and folding of human growth hormone in *Escherichia coli*. Purification and characterization. FEBS Lett 204(1), 145-150.

Bradshaw, R.A., Cancedda, F., Ericsson, L.H., Neumann, P.A., Piccoli, S.P., Schlesinger, M.J., et al. (1981). Amino acid sequence of *Escherichia coli* alkaline phosphatase. Proc Natl Acad Sci U S A 78(6), 3473-3477. doi: 10.1073/pnas.78.6.3473.

Chantalat, L., Jones, N.D., Korber, F., Navaza, J., Pavlovsky, A.G. (1995) The crystal-structure of wild-type growth-hormone at 2.5 angstrom resolution. Protein Pept.Lett. 2: 333-340. 10.2210/PDB1HGU/PDB

Chatzi, K.E., Sardis, M.F., Tsirigotaki, A., Koukaki, M., Sostaric, N., Konijnenberg, A., et al. (2017). Preprotein mature domains contain translocase targeting signals that are essential for secretion. Journal of Cell Biology 216(5), 1357-1369. doi: 10.1083/jcb.201609022.

Crane, J.M., and Randall, L.L. (2017). The Sec System: Protein Export in *Escherichia coli*. EcoSal Plus 7(2). doi: 10.1128/ecosalplus.ESP-0002-2017.

Ellis, M., Patel, P., Edon, M., Ramage, W., Dickinson, R., and Humphreys, D.P. (2017). Development of a high yielding *E. coli* periplasmic expression system for the production of humanized Fab' fragments. Biotechnol Prog 33(1), 212-220. doi: 10.1002/btpr.2393.

Engler, D.A., Matsunami, R.K., Campion, S.R., Stringer, C.D., Stevens, A., and Niyogi, S.K. (1988). Cloning of authentic human epidermal growth factor as a bacterial secretory protein and its initial structure-function analysis by site-directed mutagenesis. J Biol Chem 263(25), 12384-12390.

Gannon, P.M., Li, P., and Kumamoto, C.A. (1989). The mature portion of *Escherichia coli* maltose-binding protein (MBP) determines the dependence of MBP on SecB for export. J Bacteriol 171(2), 813-818. doi: 10.1128/jb.171.2.813-818.1989.

Guisez, Y., Fache, I., Campfield, L.A., Smith, F.J., Farid, A., Plaetinck, G., et al. (1998). Efficient secretion of biologically active recombinant OB protein (leptin) in *Escherichia coli*, purification from the periplasm and characterization. Protein Expr Purif 12(2), 249-258. doi: 10.1006/prep.1997.0836.

Hjelm, A., Soderstrom, B., Vikstrom, D., Jong, W.S., Luirink, J., and De Gier, J.W. (2015). Autotransporter-based antigen display in bacterial ghosts. Appl Environ Microbiol 81(2), 726-735. doi: 10.1128/AEM.02733-14.

Jong, W.S., Daleke-Schermerhorn, M.H., Vikstrom, D., Ten Hagen-Jongman, C.M., De Punder, K., Van Der Wel, N.N., et al. (2014). An autotransporter display platform for the development of multivalent recombinant bacterial vector vaccines. Microb Cell Fact 13, 162. doi: 10.1186/s12934-014-0162-8.

Jong, W.S., Ten Hagen-Jongman, C.M., Den Blaauwen, T., Slotboom, D.J., Tame, J.R., Wickstrom, D., et al. (2007). Limited tolerance towards folded elements during secretion of the autotransporter Hbp. Mol Microbiol 63(5), 1524-1536. doi: 10.1111/j.1365-2958.2007.05605.x.

Kaderbhai, M.A., Ugochukwu, C.C., Kelly, S.L., and Lamb, D.C. (2001). Export of cytochrome P450 105D1 to the periplasmic space of *Escherichia coli*. Appl Environ Microbiol 67(5), 2136-2138. doi: 10.1128/AEM.67.5.2136-2138.2001.

Kim, J., Rusch, S., Luirink, J., and Kendall, D.A. (2001). Is Ffh required for export of secretory proteins? FEBS Lett 505(2), 245-248.

Kujau, M.J., Hoischen, C., Riesenberg, D., and Gumpert, J. (1998). Expression and secretion of functional miniantibodies McPC603scFvDhlx in cell-wall-less L-form strains of *Proteus mirabilis* and *Escherichia coli*: a comparison of the synthesis capacities of L-form strains with an E. coli producer strain. Appl Microbiol Biotechnol 49(1), 51-58.

Kumamoto, C.A., and Beckwith, J. (1985). Evidence for specificity at an early step in protein export in *Escherichia coli*. J Bacteriol 163(1), 267-274.

Kusukawa, N., Yura, T., Ueguchi, C., Akiyama, Y., and Ito, K. (1989). Effects of mutations in heat-shock genes groES and groEL on protein export in *Escherichia coli*. EMBO J 8(11), 3517-3521.

Lam, T.L., Wong, R.S., and Wong, W.K. (1997). Enhancement of extracellular production of a *Cellulomonas fimi* exoglucanase in *Escherichia coli* by the reduction of promoter strength. Enzyme Microb Technol 20(7), 482-488.

Landeta, C., Boyd, D., and Beckwith, J. (2018). Disulfide bond formation in prokaryotes. Nat Microbiol 3(3), 270-280. doi: 10.1038/s41564-017-0106-2.

Lee, S.J., Kim, I.C., Kim, D.M., Bae, K.H., and Byun, S.M. (1998). High level secretion of recombinant staphylokinase into periplasm of *Escherichia coli*. Biotechnology Letters 20(2), 113-116. doi: 10.1023/a:1005359920522.

Loo, T., Patchett, M.L., Norris, G.E., and Lott, J.S. (2002). Using secretion to solve a solubility problem: high-yield expression in *Escherichia coli* and purification of the bacterial glycoamidase PNGase F. Protein Expr Purif 24(1), 90-98. doi: 10.1006/prep.2001.1555.

Manosroi, J., Tayapiwatana, C., Gotz, F., Werner, R.G., and Manosroi, A. (2001). Secretion of active recombinant human tissue plasminogen activator derivatives in *Escherichia coli*. Appl Environ Microbiol 67(6), 2657-2664. doi: 10.1128/AEM.67.6.2657-2664.2001.

Negoda, A., Negoda, E., and Reusch, R.N. (2010). Resolving the native conformation of *Escherichia coli* OmpA. FEBS J 277(21), 4427-4437.

Otto, B.R., Van Dooren, S.J., Nuijens, J.H., Luirink, J., and Oudega, B. (1998). Characterization of a hemoglobin protease secreted by the pathogenic *Escherichia coli* strain EB1. J Exp Med 188(6), 1091-1103. doi: 10.1084/jem.188.6.1091.

Quan, S., Hiniker, A., Collet, J.F., and Bardwell, J.C. (2013). Isolation of bacteria envelope proteins. Methods Mol Biol 966, 359-366. doi: 10.1007/978-1-62703-245-2_22.

Rathore, D., Nayak, S.K., and Batra, J.K. (1997). Overproduction of fungal ribotoxin alpha-sarcin in *Escherichia coli*: generation of an active immunotoxin. Gene 190(1), 31-35.

Ray, M.V., Meenan, C.P., Consalvo, A.P., Smith, C.A., Parton, D.P., Sturmer, A.M., et al. (2002). Production of salmon calcitonin by direct expression of a glycine-extended precursor in *Escherichia coli*. Protein Expr Purif 26(2), 249-259.

Rippmann, J.F., Klein, M., Hoischen, C., Brocks, B., Rettig, W.J., Gumpert, J., et al. (1998). Procaryotic expression of single-chain variable-fragment (scFv) antibodies: secretion in L-form cells of *Proteus mirabilis* leads to active product and overcomes the limitations of periplasmic expression in *Escherichia coli*. Appl Environ Microbiol 64(12), 4862-4869.

Robbens, J., Raeymaekers, A., Steidler, L., Fiers, W., and Remaut, E. (1995). Production of soluble and active recombinant murine interleukin-2 in *Escherichia coli*: high level expression, Kil-induced release, and purification. Protein Expr Purif 6(4), 481-486. doi: 10.1006/prep.1995.1064.

Rua, M.L., Atomi, H., Schmidt-Dannert, C., and Schmid, R.D. (1998). High-level expression of the thermoalkalophilic lipase from *Bacillus thermocatenulatus* in *Escherichia coli*. Appl Microbiol Biotechnol 49(4), 405-410.

Schibich, D., Gloge, F., Pohner, I., Bjorkholm, P., Wade, R.C., Von Heijne, G., et al. (2016). Global profiling of SRP interaction with nascent polypeptides. Nature 536(7615), 219-223. doi: 10.1038/nature19070.

Schierle, C.F., Berkmen, M., Huber, D., Kumamoto, C., Boyd, D., and Beckwith, J. (2003). The DsbA signal sequence directs efficient, cotranslational export of passenger proteins to the *Escherichia coli* periplasm via the signal recognition particle pathway. J Bacteriol 185(19), 5706-5713. doi: 10.1128/jb.185.19.5706-5713.2003.

Schlegel, S., Rujas, E., Ytterberg, A.J., Zubarev, R.A., Luirink, J., and De Gier, J.W. (2013). Optimizing heterologous protein production in the periplasm of *E. coli* by regulating gene expression levels. Microb Cell Fact 12, 24. doi: 10.1186/1475-2859-12-24.

Sijbrandi, R., Urbanus, M.L., Ten Hagen-Jongman, C.M., Bernstein, H.D., Oudega, B., Otto, B.R., et al. (2003). Signal recognition particle (SRP)-mediated targeting and Sec-dependent translocation of an extracellular *Escherichia coli* protein. J Biol Chem 278(7), 4654-4659. doi: 10.1074/jbc.M211630200.

Sivakesava, S., Xu, Z.N., Chen, Y.H., Hackett, J., Huang, R.C., Lam, E., Lam, T.L., Siu, K.L Wong, R.S.C., Wong, W.K.R. (1999). Production of excreted human epidermal growth factor (hEGF) by an efficient recombinant *Escherichia coli* system. Process Biochemistry 34(9), doi:10.1016/S0032-9592(99)00013-8.

Slos, P., Speck, D., Accart, N., Kolbe, H.V., Schubnel, D., Bouchon, B., et al. (1994). Recombinant cholera toxin B subunit in *Escherichia coli*: high-level secretion, purification, and characterization. Protein Expr Purif 5(5), 518-526. doi: 10.1006/prep.1994.1071.

Soares, C.R., Gomide, F.I., Ueda, E.K., and Bartolini, P. (2003). Periplasmic expression of human growth hormone via plasmid vectors containing the lambdaPL promoter: use of HPLC for product quantification. Protein Eng 16(12), 1131-1138. doi: 10.1093/protein/gzg114.

Sone, M., Kishigami, S., Yoshihisa, T., and Ito, K. (1997). Roles of disulfide bonds in bacterial alkaline phosphatase. J Biol Chem 272(10), 6174-6178. doi: 10.1074/jbc.272.10.6174.

Stec, B., Holtz, K.M., and Kantrowitz, E.R. (2000). A revised mechanism for the alkaline phosphatase reaction involving three metal ions. J Mol Biol 299(5), 1303-1311. doi: 10.1006/jmbi.2000.3799.

Steiner, D., Forrer, P., Stumpp, M.T., and Pluckthun, A. (2006). Signal sequences directing cotranslational translocation expand the range of proteins amenable to phage display. Nat Biotechnol 24(7), 823-831. doi: 10.1038/nbt1218.

Sugawara, E., and Nikaido, H. (1992). Pore-forming activity of OmpA protein of *Escherichia coli*. J Biol Chem 267(4), 2507-2511.

Tanaka, T., Horio, T., and Matuo, Y. (2002). Secretory production of recombinant human C-reactive protein in *Escherichia coli,* capable of binding with phosphorylcholine, and its characterization. Biochem Biophys Res Commun 295(1), 163-166.

Tong, L., Lin, Q., Wong, W.K., Ali, A., Lim, D., Sung, W.L., et al. (2000). Extracellular expression, purification, and characterization of a winter flounder antifreeze polypeptide from *Escherichia coli*. Protein Expr Purif 18(2), 175-181. doi: 10.1006/prep.1999.1176.

Van Den Berg Van Saparoea, H.B., Houben, D., De Jonge, M.I., Jong, W.S.P., and Luirink, J. (2018). Display of Recombinant Proteins on Bacterial Outer Membrane Vesicles by Using Protein Ligation. Appl Environ Microbiol 84(8). doi: 10.1128/AEM.02567-17.

Wan, E.W., and Baneyx, F. (1998). TolAIII co-overexpression facilitates the recovery of periplasmic recombinant proteins into the growth medium of *Escherichia coli*. Protein Expr Purif 14(1), 13-22. doi: 10.1006/prep.1998.0941.

Wen, C., Gan, R., and Zhu, S. (2003). Construction of Secretory Expression System Suitable to Express Glucagon Under the Control of P L Promoter. Current Microbiology 47(3), 180-185. doi: 10.1007/s00284-002-3988-y.

Winter, J., Neubauer, P., Glockshuber, R., and Rudolph, R. (2001). Increased production of human proinsulin in the periplasmic space of *Escherichia coli* by fusion to DsbA. J Biotechnol 84(2), 175-185.

Xu, R., Du, P., Fan, J.J., Zhang, Q., Li, T.P., and Gan, R.B. (2002). High-level expression and secretion of recombinant mouse endostatin by *Escherichia coli*. Protein Expr Purif 24(3), 453-459. doi: 10.1006/prep.2001.1585.

Yang, J., Moyana, T., Mackenzie, S., Xia, Q., and Xiang, J. (1998). One hundred seventy-fold increase in excretion of an FV fragment-tumor necrosis factor alpha fusion protein (sFV/TNF-alpha) from *Escherichia coli* caused by the synergistic effects of glycine and triton X-100. Appl Environ Microbiol 64(8), 2869-2874.

Zhang, H., Li, Z., Qian, Y., Zhang, Q., Du, P., Gan, R., et al. (2007). Cultivation of recombinant *Escherichia coli* for secretory production of human epidermal growth factor under control of PL promoter. Enzyme and Microbial Technology 40(4), 708-715. doi: 10.1016/j.enzmictec.2006.06.001.

Zhang, W., Lu, J., Zhang, S., Liu, L., Pang, X., and Lv, J. (2018). Development an effective system to expression recombinant protein in *E. coli* via comparison and optimization of signal peptides: Expression of *Pseudomonas fluorescens* BJ-10 thermostable lipase as case study. Microb Cell Fact 17(1), 50. doi: 10.1186/s12934-018-0894-y.

Zhou, Y., Liu, P., Gan, Y., Sandoval, W., Katakam, A.K., Reichelt, M., et al. (2016). Enhancing full-length antibody production by signal peptide engineering. Microb Cell Fact 15, 47. doi: 10.1186/s12934-016-0445-3.
